# Targeting *LMO2*-induced autocrine FLT3 signaling to overcome chemoresistance in early T-cell precursor acute lymphoblastic leukemia

**DOI:** 10.1038/s41375-024-02491-5

**Published:** 2025-01-23

**Authors:** Cedric S. Tremblay, Jesslyn Saw, Feng Yan, Jacqueline A. Boyle, Ovini Amarasinghe, Shokoufeh Abdollahi, Anh N. Q. Vo, Benjamin J. Shields, Chelsea Mayoh, Hannah McCalmont, Kathryn Evans, Anna Steiner, Kevin Parsons, Matthew P. McCormack, David R. Powell, Nicholas C. Wong, Stephen M. Jane, Richard B. Lock, David J. Curtis

**Affiliations:** 1 Department of Immunology, Max Rady College of Medicine, University of Manitoba, Winnipeg, MB Canada; 2 Paul Albrechtsen Research Institute CancerCare Manitoba, Winnipeg, MB Canada; 3 Children’s Hospital Research Institute of Manitoba (CHRIM), Winnipeg, MB Canada; 4 Australian Centre for Blood Diseases (ACBD), School of Translational Medicine, Monash University, Melbourne, VIC Australia; 5 Bioinformatics Division, Walter and Eliza Hall Institute (WEHI) of Medical Research, Parkville, VIC Australia; 6 Children’s Cancer Institute, Lowy Cancer Research Centre, School of Clinical Medicine, UNSW Medicine & Health, UNSW Centre for Childhood Cancer Research, UNSW Sydney, Sydney, NSW Australia; 7 Community and Researcher Engagement (CaRE) program, School of Translational Medicine, Monash University, Melbourne, VIC Australia; 8 Women in Lymphoma, Lymphoma Australia, Brisbane, QLD Australia; 9 Monash Bioinformatics Platform, Monash University, Clayton, VIC Australia; 10 Department of Medicine, School of Translational Medicine, Monash University, Melbourne, VIC Australia; 11 Department of Clinical Haematology, Alfred Hospital, Prahran, VIC Australia

**Keywords:** Acute lymphocytic leukaemia, Cancer stem cells, Cell signalling, Preclinical research

## Abstract

Early T-cell Precursor Acute Lymphoblastic Leukemia (ETP-ALL) is an immature subtype of T-cell acute lymphoblastic leukemia (T-ALL) commonly show deregulation of the LMO2-LYL1 stem cell transcription factors, activating mutations of cytokine receptor signaling, and poor early response to intensive chemotherapy. Previously, studies of the *Lmo2* transgenic mouse model of ETP-ALL identified a population of stem-like T-cell progenitors with long-term self-renewal capacity and intrinsic chemotherapy resistance linked to cellular quiescence. Here, analyses of *Lmo2* transgenic mice, patient-derived xenografts, and single-cell RNA-sequencing data from primary ETP-ALL identified a rare subpopulation of leukemic stem cells expressing high levels of the cytokine receptor FLT3. Despite a highly proliferative state, these FLT3-overexpressing cells had long-term self-renewal capacity and almost complete resistance to chemotherapy. Chromatin immunoprecipitation and assay for transposase-accessible chromatin sequencing demonstrated FLT3 and its ligand may be direct targets of the LMO2 stem-cell complex. Media conditioned by *Lmo2* transgenic thymocytes revealed an autocrine FLT3-dependent signaling loop that could be targeted by the FLT3 inhibitor gilteritinib. Consequently, gilteritinib impaired in vivo growth of ETP-ALL and improved the sensitivity to chemotherapy. Furthermore, gilteritinib enhanced response to the BCL2 inhibitor venetoclax, which may enable “chemo-free” treatment of ETP-ALL. Together, these data provide a cellular and molecular explanation for enhanced cytokine signaling in *LMO2*-driven ETP-ALL beyond activating mutations and a rationale for clinical trials of FLT3 inhibitors in ETP-ALL.

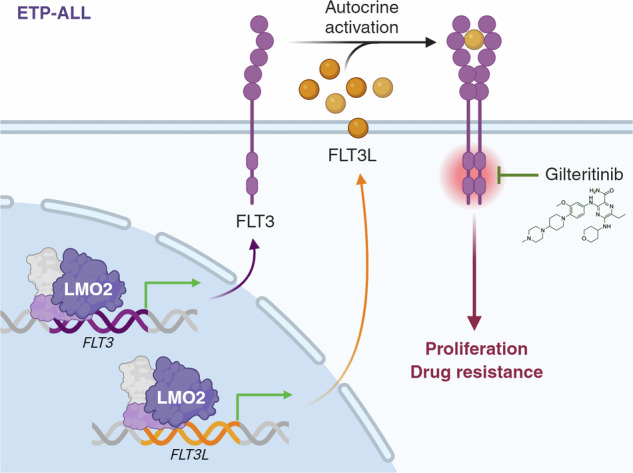

## Introduction

The concept of a rare population of leukemia stem cells (LSCs) with stem-cell like properties including self-renewal and drug resistance was first validated decades ago in acute myeloid leukemia (AML) [1, 2] and more recently in acute lymphoblastic leukemia (ALL) [3, 4]. These properties enable LSCs to initiate leukemia when transplanted in conditioned animals and lead to relapse following chemotherapy [1, 5].

Genomic studies of matched diagnosis and relapse bone marrow (BM) samples have shown that relapse arises from either LSCs present in the diagnostic sample or ancestral clones of so-called preleukemic stem cells (preLSCs) that only harbor some but not all of the mutations found in the tumor at diagnosis [6–8]. PreLSCs emerge following the acquisition of initiating mutations in hematopoietic stem and progenitor cells (HSPCs) that can be tracked from the early stage of the disease [8]. Clonal evolution of preLSCs—through acquisition of cooperative mutations—drives transformation into LSCs that are responsible for leukemia aggressiveness and dissemination to secondary organs [9–11]. The persistence of LSCs following induction chemotherapy has been shown to be an important prognostic factor in AML and B-cell ALL (B-ALL) [5, 12–14]. LSCs are less well defined in T-cell ALL (T-ALL) [3, 15–17], a heterogenous group of aggressive leukemias treated with high-dose induction chemotherapy followed by prolonged maintenance regimens [18, 19].

A better understanding of the LSC populations in T-ALL may help identify new strategies to improve cure rates without the need for intensive and prolonged chemotherapy. This goal is of utmost importance for the early T-cell precursor (ETP-ALL) subtype, which is characterized by an immature T-cell immunophenotype with stem and/or myeloid cell markers and a distinct mutational profile [7, 10]. ETP-ALL is intrinsically resistant to chemotherapy, although outcomes are similar to other types of T-ALL if high-dose therapy can be delivered [20, 21]. The explanation for chemoresistance of ETP-ALL is unknown but may reflect the immature, stem cell-like phenotype of this aggressive malignancy.

Genomic profiling showed that ETP-ALL cases commonly exhibit dysregulation of the stem cell transcription factors LMO2 and LYL1 [22, 23]. Accordingly, transgenic or retroviral overexpression of LMO2 leads to T-ALL with a transcriptional profile resembling ETP-ALL [24]. In these murine models, aberrant expression of LMO2 initiates leukemia by reprogramming a fraction of T-cell progenitors into preLSCs many months before the development of overt T-ALL [25, 26]. Studies using these mice have shown the importance of cell cycle quiescence and signal transducer and activator of transcription 5 (STAT5) signaling for self-renewal and chemoresistance of preLSCs [24, 27, 28]. Clonal evolution of these preLSCs through the acquisition of cooperative mutations leads to T-ALL, which contains LSCs with a more heterogeneous immunophenotype [29, 30].

Although the mechanisms allowing LSCs to withstand high-dose chemotherapy and drive relapse remain unclear, signaling molecules secreted by the microenvironment (e.g., cytokines, chemokines, growth factors) supporting key stem-like properties of LSCs have been shown to promote drug resistance [31, 32]. Crucial signaling molecules regulating hematopoietic stem cell (HSC) function and differentiation into different blood cell lineages, such as interleukin-7 receptor (IL-7R) and NOTCH1, are frequently mutated in all subtypes of T-ALL [19, 22, 33, 34]. In contrast, activating mutations of FMS-like tyrosine kinase 3 (FLT3) are almost exclusively found in ETP-ALL [19, 35–38], suggesting a role for aberrant FLT3 signaling in this high-risk subtype of T-ALL.

Here we used relevant models of *LMO2*-driven ETP-ALL to show that high expression of FLT3 defines a subpopulation of chemoresistant preLSCs that expand and evolve into LSCs during leukemogenesis. We also demonstrate that this FLT3-overexpressing subset of chemoresistant LSCs can be effectively targeted by the FLT3 inhibitor gilteritinib in human ETP-ALL.

## Results

### Identification of aberrant FLT3-overexpressing subpopulation in preleukemic thymocytes

**Fig. 1:**
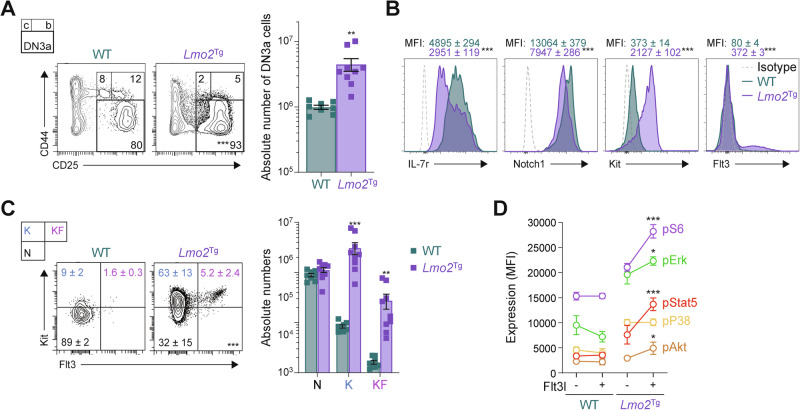
Aberrant response to Flt3l in preleukemic *Lmo2*-transgenic DN3a thymocytes. **A** Representative flow cytometric analysis of DN3 thymocyte subsets (DN3a, b, and c) in 6-week old wild-type (WT) and *Lmo2*-transgenic (*Lmo2*^Tg^) mice. Median, 2-way ANOVA with Šídák correction test (DN3 subsets; complete data in Fig. S1A) and Student’s *t*-test (absolute number of DN3a cells); **P < 0.01, ***P < 0.001 compared to WT mice. **B** Levels of Il-7r, Notch1, Kit (CD117), and Flt3 (CD135) at the surface of DN3a T-cell progenitors from 6-week old WT and *Lmo2*^Tg^ mice. Mean fluorescence intensity (MFI) ± S.E.M. of *N* > 5 individual mice. **C** Representative flow cytometry analysis (left) and absolute numbers (right) of DN3a thymocyte subpopulations in the thymus of 6-week old WT and *Lmo2*^Tg^ mice, assessed by flow cytometry using the Kit and Flt3 surface markers. N (Kit^-^Flt3^-^), K (Kit^+^Flt3^low^), and KF (Kit^hi^Flt3^hi^) populations are indicated. Median ± S.E.M., 2-way ANOVA with Tukey’s correction test; **P < 0.01, ***P < 0.001 compared to WT mice. **D** Levels of activated Akt (pAkt), Erk (pErk), P38 (pP38), S6 (pS6), and Stat5 (pStat5) in DN3a thymocytes from 6-week-old mice stimulated with Flt3-ligand (Flt3l). MFI ± S.E.M. of *N* = 3 biological replicates are shown, ordinary 1-way ANOVA with Tukey’s correction test; *P < 0.05, **P < 0.01, ***P < 0.001 compared to basal levels.

In the *CD2-Lmo2* transgenic (*Lmo2*^Tg^) model of ETP-ALL, leukemia arises from an expanded CD4^-^CD8^-^CD44^-^CD25^+^CD28^-^ (DN3a) thymocyte population (Figs. 1A and S1A), which contains preLSCs that display a stem-like phenotype [24–26, 29]. To further immunophenotypically define these preLSCs, we assessed the expression of hematopoiesis-regulating receptors, such as Il-7r, Notch1, Kit, and Flt3, which have been implicated in leukemia development [29, 39]. As previously reported [40], surface expression of Kit was homogenously increased in preLSCs (Figs. 1B and S1B). In contrast, cell surface expression of Il-7r, and Notch1 were homogeneously decreased. Most notable was a discrete subpopulation expressing high levels of Flt3 (Fig. 1B). Co-staining for Kit and Flt3 defined 3 sub-populations within the preleukemic DN3a fraction (Fig. 1C): N (Kit^−^Flt3^−^), K (Kit^+^Flt3^low^) and KF (Kit^hi^Flt3^hi^). In 2-month-old *Lmo2*^Tg^ mice, the DN3a^K^ and DN3a^KF^ populations were expanded 37- and 23-fold but only constituted 1 in 50 and 1 in 700 thymocytes, respectively. In contrast, numbers of DN3a^N^ thymocytes were not increased, and proportionally decreased compared with wild-type mice (Fig. 1C). To assess the impact on FLT3 signaling, we performed in vitro stimulation of fluorescence-activated cell sorting (FACS)-isolated DN3a cells with Flt3-ligand (Flt3l). Flt3l activated canonical effectors from multiple signaling pathways including JAK-STAT (phosphorylated Stat5; pStat5), MAP kinase (pErk), and mTOR (pS6, pAkt) pathways (Fig. 1D). Altogether, our results revealed an expanded subpopulation of preleukemic DN3a thymocytes that express high levels of Flt3.

### FLT3-overexpression defining proliferative preLSCs with long-term self-renewal capacity

**Fig. 2:**
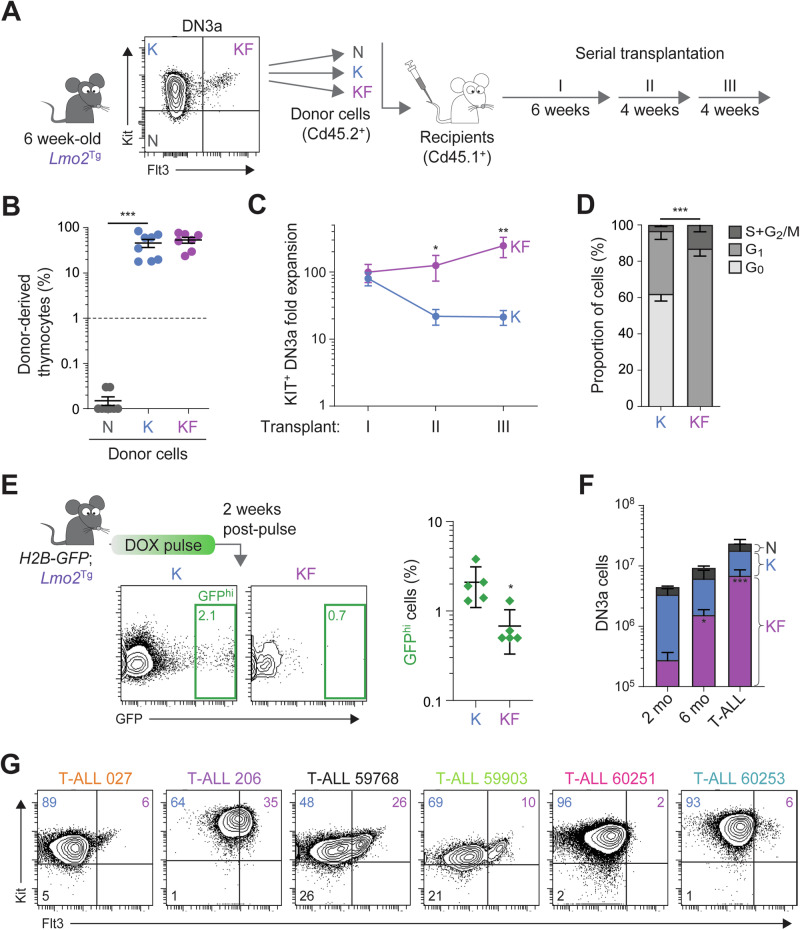
Flt3 overexpression associated with proliferation and expansion of preLSCs. **A** Scheme for serial transplantation of purified N, K, and KF DN3a T-cell progenitor populations into primary (I), secondary (II) and tertiary (III) recipients. **B** Repopulation capacity of donor-derived N, K, and KF populations of DN3a thymocytes enumerated in the thymus of primary recipient mice. Proportion (%) of donor-derived thymocytes, Median ± 95% CI, ordinary 1-way ANOVA with Tukey’s correction test. Minimal repopulation capacity (fold of 1) is indicated by a dashed line. **C** Fold expansion of donor-derived *Lmo2*-transgenic K and KF populations of DN3a thymocytes enumerated in the thymus of primary (I), secondary (II), and tertiary (III) recipients. Mean ± S.E.M., 2-way ANOVA with Tukey’s correction test; *P < 0.05 and **P < 0.01 compared to K donor cells. **D** Cell-cycle analysis in subpopulations of DN3a thymocytes from preleukemic *Lmo2*^Tg^ mice. Mean ± S.E.M., Student’s *t* test; ***P < 0.001. **E** Scheme for H2B-GFP labeling followed by 2 weeks of chase without Doxycycline, with representative flow cytometric analysis of GFP expression in subpopulations of DN3a thymocytes from *H2B-GFP; Lmo2*^Tg^ mice. Populations retaining high levels of H2B-GFP labeling (GFP^hi^) are framed, with the average proportion (%) indicated. Mean ± S.E.M., 2-way ANOVA with Tukey’s correction test; *P < 0.05 and ***P < 0.001. **F** Absolute numbers of DN3a T-cell populations in the thymus of *Lmo2*^Tg^ mice at 2 months, 6 months, and at overt T-ALL. Median ± S.E.M., 2-way ANOVA with Tukey’s correction test; *P < 0.05 and ***P < 0.001. **G** Representative flow cytometry analysis of DN3a thymocyte subpopulations in primary *Lmo2*^Tg^ leukemias. Average proportion of N, K, and KF populations are indicated for each T-ALL.

We performed serial transplants of FACS-isolated N, K, and KF subpopulations of preleukemic DN3a cells to determine their self-renewal capacity (Fig. 2A). In primary recipients, both DN3a^K^ and DN3a^KF^ thymocyte populations repopulated the thymus of sublethally-irradiated recipients with comparable efficacy, whilst DN3a^N^ cells were devoid of repopulating ability (Figs. 2B and S2A). However, serial transplantation revealed increased self-renewal capacity of DN3a^KF^ cells compared to DN3a^K^ cells (Figs. 2C and S2B). We examined cell cycle of DN3a^KF^ to determine if they were more quiescent, a feature of HSCs associated with long-term self-renewal [41, 42]. Paradoxically, DN3a^KF^ cells were all in active cell cycle (G_1_, S, and G_2_/M) compared with DN3a^K^ cells, where more than half were in G_0_ (Fig. 2D). To confirm the proliferative nature of DN3a^KF^ cells, we used the *TetOP-H2B-GFP*^KI/+^;*Lmo2*^Tg^ (*H2B-GFP*;*Lmo2*^Tg^) mouse model, which we have previously used to describe the cell cycle kinetics of preLSCs [24]. As previously reported [24, 28], 2% of the total DN3a thymocytes remained quiescent, as defined by retention of H2B-GFP (GFP^hi^) for at least 2 weeks after withdrawal of doxycycline following a 6-week labeling period (Fig. S2C). We observed a similar proportion of GFP^hi^ cells in the DN3a^K^ subpopulation (2.1 ± 0.5%), which represents the majority of *Lmo2*^Tg^ DN3a thymocytes (Fig. 2E). In contrast, 3-fold fewer DN3a^KF^ thymocytes retained GFP labeling (0.7 ± 0.2%), indicating that this subpopulation is more proliferative.

To determine the fate of DN3a^KF^ cells during disease progression, we compared numbers of DN3a subpopulations in 2-, 6-month old and leukemic *Lmo2*^Tg^ mice. Analysis of ageing mice revealed a 25-fold increase from 2-month old mice to overt T-ALL (Figs. 2F and S2D), which was associated with progressive *Flt3* expression increase in the bulk DN3a population (Fig. S2E). DN3a^KF^ thymocytes expanded 3.7 and 6.9 times more than DN3a^K^ thymocytes in 6-month old and leukemic *Lmo2*^Tg^ mice, respectively. Accordingly, DN3a^KF^ cells were present in all primary T-ALL analyzed (Fig. 2G), irrespective of their immunophenotypical features (Fig. S2F), However, the Flt3 expression profile was more variable in leukemic *Lmo2*^Tg^ mice compared to what we observed during the pre-leukemic phase. Overall, our data show that preLSCs expressing high levels of Flt3 display increased long-term self-renewal capacity and proliferation associated with progressive expansion during leukemia development.

### FLT3 overexpression is linked to leukemia repopulating activity in human ETP-ALL

**Fig. 3:**
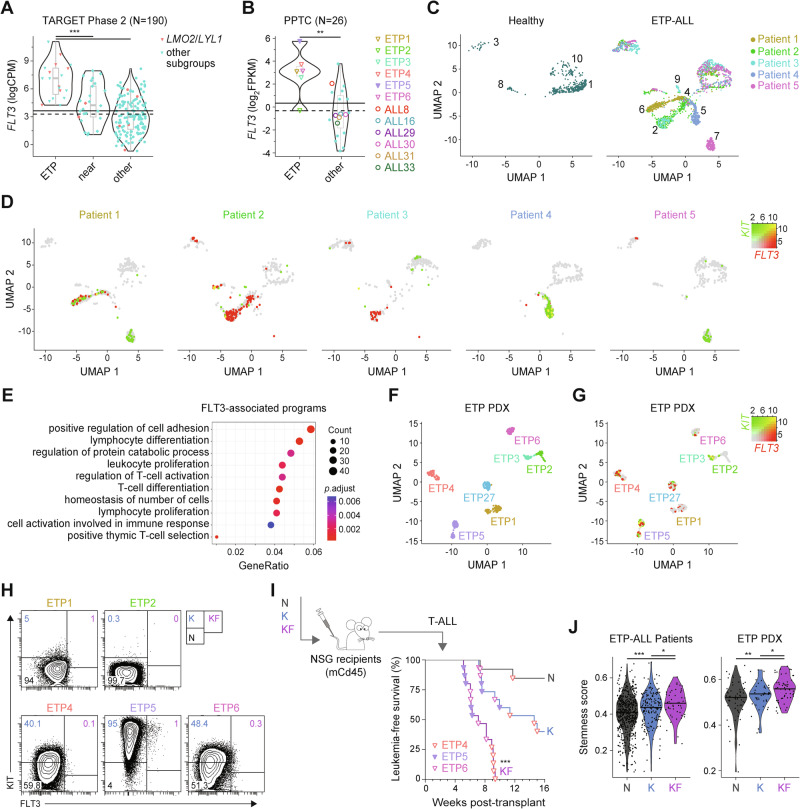
LSCs enriched in KF population from human ETP-ALL*.* Expression levels of *FLT3* in primary T-ALL samples from the **A** TARGET-ALL Phase 2 and **B** PPTC cohorts. Immunophenotypically-defined subtypes of T-ALL listed in the TARGET-ALL Phase 2 cohort as ETP, near-ETP (near) and more mature T-ALL subtypes (other). CPM counts per million mapped reads, FPKM fragments per kilo base of transcript per million mapped fragments, PPTC Pediatric Preclinical Testing Consortium. Solid line: mean; dashed line: median. ^#^Wilcox *P* = 0.019; Student’s *t* test *P* < 0.05 compared to other subgroups. Two-dimension uniform manifold approximation, and projection (UMAP) of the processed single-cell RNA-seq gene expression data from 4–5 healthy individuals and 5 patients with refractory/relapsed ETP-ALL visualized in (**C**) distinct patient-specific clusters along with heterogeneous clusters, and (**D**) log-normalized *FLT3* and *KIT* expression in these clusters for healthy donors (merged) and individual ETP-ALL patients. Expression of *KIT* in green and *FLT3* in red, with co-expression in yellow (KF cells). **E** Gene Ontology (GO) enrichment of the top 500 differentially-expressed genes (DEGs) in KF compared to K cells from all ETP-ALL patients analyzed. The size of the circle represents the number of genes enriched in the pathway, the color of the circle represents the adjusted *P* value. Two-dimension UMAP visualization of single-cell RNA-seq data from 7 individual PDX of human ETP-ALL of (**F**) color-coded tumor cell clusters and (**G**) log-normalized *FLT3* and *KIT* expression in these clusters. **H** Representative flow cytometry analysis of subpopulations of tumor cells in individual PDX of human ETP-ALL. Average proportion of N, K, and KF populations are indicated for each PDX models. **I** Flow sorting and schematic representation of the transplantation strategy of purified leukemic subpopulation of ETP4, ETP5, and ETP6 cells (top). Kaplan-Meier curves of mice injected with the purified populations (bottom). Log-rank (Mantel-Cox) test; ***P < 0.001 compared to K and N donor cell populations. **J** Stemness score inferred from the *LMO2*-associated preLSC gene signature for each tumor cell subpopulation in refractory/relapsed ETP-ALL samples and ETP PDXs.

Aberrant expression of FLT3 is found in one-third of T-ALL cases, especially in ETP-ALL [43, 44]. We examined *FLT3* mRNA levels in the TARGET-ALL Phase2 cohort, which can be separated according to ETP, near-ETP, and other more mature T-ALL immunophenotypes [19]. Expression of *FLT3* was significantly elevated in ETP-ALL, in particular within the *LMO2*/*LYL1* subgroup (Figs. 3A and S3A). Consistent with the TARGET-ALL Phase2 cohort, transcriptional profiling of patient-derived xenografts (PDX) obtained from the Pediatric Preclinical Testing Consortium (PPTC) [45–47] showed that ETP-ALL displayed the highest *FLT3* expression with elevated mRNA levels in 5 out of 6 ETP but only 1 out of 19 other more mature T-ALL samples (Fig. 3B).

Given that the above data were generated on bulk leukemic samples, we explored publicly available single-cell RNA-sequencing (scRNA-seq) data to decipher the role of FLT3 overexpression in human ETP-ALL [48]. Uniform manifold approximation and projection (UMAP) of scRNA-seq profiling of 5 relapsed or refractory ETP-ALL patients and 4 healthy donors revealed 10 distinct cell clusters (Fig. S3B). The majority of these clusters (2, 4, 5, 6, 7, 9) were specific to individual ETP-ALL patients, while the remaining 4 clusters (1, 3, 8, 10) were shared with healthy donors corresponding to B cells, mature T cells and monocytes (Figs. 3C and S3B, C) and thus, were excluded from further analyses. Analysis of differentially-expressed genes (DEGs) combining ImmGen [49] and well-established lineage markers showed that the majority of cells from ETP-ALL clusters expressed hematopoietic stem and progenitor signatures (Figs. S4–5). Analysis of *KIT* and *FLT3* expression demonstrated variable frequency of KF in 4 of 5 ETP-ALL cases (Figs. 3D and S6A), while KF cells were absent in healthy donors (Fig. S6A, B). Gene ontology (GO) enrichment and Reactome pathway analyses of the top 1,000 DEGs between KF and K cells linked *FLT3* overexpression with cell adhesion, T-cell activation, and proliferation (Fig. 3E), as well as interferon signaling (Fig. S6C). Expression of cell-cycle specific genes suggested KF cells were more frequently in S and G2/M phase (Fig. S6D), thereby linking high levels of FLT3 with tumor cell proliferation in relapsed/refractory ETP-ALL.

We next used PDX models of ETP-ALL samples harvested at time of diagnosis (Fig. 3B) to explore the functional relevance of KF cells. This cohort included one ETP-ALL (ETP5) with FLT3-activating mutation (D835Y; Fig. S7A) [27]. Visualization of scRNA-seq data from these samples revealed distinct cell clusters for individual ETP-ALL with high intrapatient correlation (Fig. 3F). Gene expression (Fig. 3G) and flow cytometry (Fig. 3H) profiling demonstrated variable frequency of KF cells in the 5 ETP-ALL PDX analyzed (Fig. S7B). These KF cells displayed a proliferative signature with around 70% in the S and G2/M phases of the cell cycle (Fig. S7C). GO enrichment and pathway analyses of the top 500 DEGs in KF cells revealed a proliferative and inflammatory-related transcriptional profile (Fig. S7D, E) similar to tumor cells expressing high levels of FLT3 in primary relapsed/refractory ETP-ALL.

To assess the leukemia repopulating activity of KF cells, we transplanted equal numbers of FACS-isolated N, K, and KF cells from 3 individual ETP-ALL PDX (Fig. 3I). Consistent with the *Lmo2*^Tg^ mouse experiments, KF had the greatest leukemia repopulating activity, with all recipients developing overt leukemia within 10 weeks (Fig. 3I). We next assessed whether high FLT3 expression in ETP-ALL tumors correlate with stemness using the *LMO2*-associated preLSC gene signature we previously defined [24]. When compared to N and K fractions, KF cells from both ETP-ALL patients and ETP PDX samples displayed higher stemness scores (Fig. 3J). Thus, FLT3 overexpression defines an aggressive subpopulation of proliferative LSCs in human ETP-ALL characterized by molecular programs related to stemness and inflammatory response.

### FLT3 overexpression defines a subset of chemoresistant preleukemic and leukemic repopulating cells

**Fig. 4:**
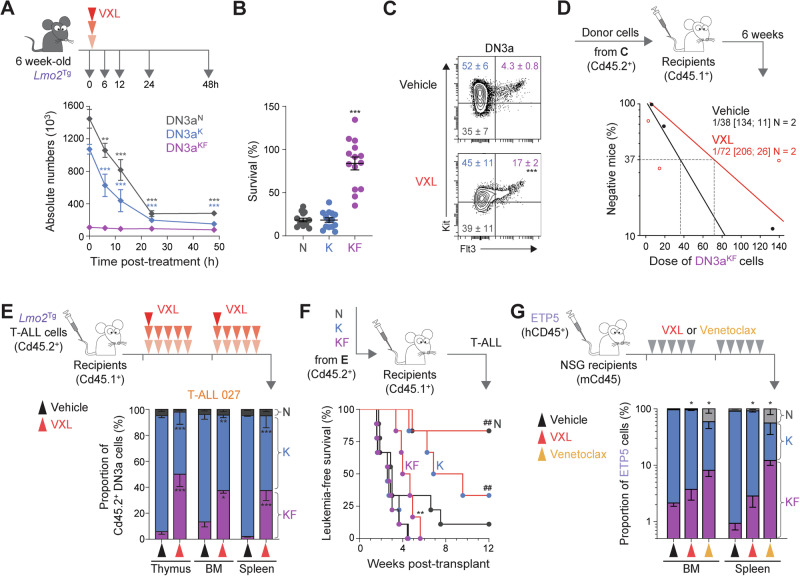
Chemoresistance of FLT3-overexpressing preleukemic and leukemic repopulating cells. **A** Treatment schematic and absolute numbers of DN3a thymocyte subpopulations in the thymus of 6-week old *Lmo2*^Tg^ mice at the indicated time following administration of induction-like therapy for T-ALL (VXL: vincristine, dexamethasone, and *L*-asparaginase) [50]. Mean ± S.E.M., 2-way ANOVA with Tukey’s correction test; **P < 0.01 and ***P < 0.001 compared to untreated (0 h post-treatment). Survival (**B**) and representative flow cytometry analysis (**C**) of the DN3a T-cell progenitor subpopulations in the thymus of 6-week old *Lmo2*^Tg^ mice, at 24 h following administration of vehicle or VXL chemotherapy. For survival analyses: Median ± S.E.M., ordinary 1-way ANOVA with Tukey’s correction test; ***P < 0.001 compared to other subpopulations. For the proportion of DN3a subpopulations: Median ± S.E.M., 2-way ANOVA with Tukey’s correction test; ***P < 0.001 compared to vehicle. **D** *P*reLSC frequency within the DN3a^KF^ thymocyte population of *Lmo2*^Tg^ mice treated with vehicle or VXL chemotherapy assessed by limiting dilution assays. Mice were scored positive when T-cell lineage reconstitution was >1%, as previously described [101]. PreLSC frequencies [95% confidence intervals] were calculated from 2 biological replicates. **E** Experimental setting for assessing the therapy-resistance of leukemic DN3a subpopulations in sublethally-irradiated Cd45.1^+^ recipients injected with *Lmo2*^Tg^ primary T-ALL, treated with either vehicle and VXL chemotherapy (top) and proportion of leukemia-derived DN3a cell subpopulations in the thymus, bone marrow (BM) and spleen of recipients transplanted with the primary *Lmo2*^Tg^ T-ALL 027, harvested 24 h after the last administration of VXL chemotherapy (bottom). **F** Schematic representation of the transplantation strategy of 5 × 104 purified leukemic DN3a subpopulations of T-ALL 027 cells harvested from the thymus of recipients, 24 h after the last administration of either vehicle or VXL chemotherapy into sublethally-irradiated secondary Cd45.1^+^ recipients (top). Kaplan-Meier curves of mice injected with the purified populations (bottom; Vehicle-treated DN3a^N^, *N* = 9; Vehicle-treated DN3a^K^, *N* = 9; Vehicle-treated DN3a^KF^, *N* = 9; VXL-treated DN3a^N^, *N* = 6; VXL-treated DN3a^K^, *N* = 6; VXL-treated DN3a^KF^, *N* = 6). Log-rank (Mantel-Cox) test; **P < 0.01 compared to Vehicle-treated DN3a^KF^; ^##^*P* < 0.01 compared to VXL-treated DN3a^KF^. **G** Experimental setting for testing the effect of VXL chemotherapy and venetoclax monotherapy on tumor burden in the ETP5 PDX model (top), and proportion of ETP5 cell subpopulations in the bone marrow (BM) and spleen of recipients harvested 24 h after the last dose of treatment (bottom). Sublethally-irradiated NSG recipients were transplanted with ETP5 cells, randomized, and subsequently treated when the average proportion of human leukemic cells reached 1% in the peripheral blood. Mean ± S.E.M., 2-way ANOVA with Tukey correction test; *P < 0.05 compared to vehicle.

Using the *H2B-GFP* mouse model, we previously demonstrated an important role for slow cell cycle in chemoresistance of preLSCs [24, 28]. To assess the chemoresistance of proliferative KF cells, we enumerated preleukemic DN3a populations following a single dose of chemotherapy – a combination of vincristine, dexamethasone, and *L*-asparaginase (VXL) [50]. Time course analysis showed an 80% reduction of DN3a^N^ and DN3a^K^ thymocytes within the first 24 h after chemotherapy (Figs. 4A, B and S8A). In contrast, numbers of DN3a^KF^ thymocytes were reduced by less than 20%. This differential resistance of *Lmo2*^Tg^ DN3a thymocytes according to Flt3 expression was similar to the difference in chemoresistance of BM HSCs (Lin^-^Sca-1^+^Kit^+^CD150^+^CD48^-^) compared with more mature Lin^-^Kit^+^ (LK) progenitors (Fig. S8B). Consequently, 24-h after chemotherapy there was a 4-fold enrichment of DN3a^KF^ cells (Figs. 4C and S8C). We performed a limiting-dilution transplantation assay to determine the functional impact of chemotherapy on preleukemic KF cells. The frequency of repopulating cells was not significantly different in DN3a^KF^ thymocytes from chemotherapy-treated *Lmo2*^Tg^ mice (Fig. 4D), with 3 out of 9 recipients developing overt T-ALL by the time of analysis (Fig. S8D). Thus, Flt3-overexpressing preLSCs are profoundly chemoresistant despite their proliferative nature.

To examine the chemoresistance of KF cells in established leukemia, mice engrafted with 3 independent primary *Lmo2*^Tg^-derived immature/ETP-ALL (T-ALL 027, 206, and 60251; Figs. 4E and S8E) were treated with VXL chemotherapy or vehicle for 2 weeks, and tumor burden was assessed in the thymus, BM and spleen 24 h after the last dose. At this time point, 2 of the 3 leukemias showed enrichment of leukemic DN3a^KF^ cells in all hematopoietic organs analyzed (Figs. 4E and S8F), with the average proportion of surviving cells more than 5-fold higher in DN3a^KF^ thymocytes (Fig. S8G). Transplant of these therapy-treated populations showed that leukemogenicity was maintained in leukemic DN3a^KF^ cells but markedly reduced for DN3a^N^ and DN3a^K^ cells (Fig. 4F).

We next assessed whether FLT3-overexpressing cells are chemoresistant in human ETP-ALL, by measuring the numbers of KF cells at 24 h after VXL chemotherapy in mice engrafted with PDX ETP-5 (Fig. 4G). Consistent with the mouse data, analysis 24 h after the last dose of VXL chemotherapy showed a 2 to 3-fold enrichment of KF cells in the BM and spleen, respectively. We also examined FLT3-overexpressing cells following treatment with venetoclax, a potent and highly-selective BCL2 inhibitor clinically active in patients with relapsed/refractory ETP-ALL [51–53]. Resistance to venetoclax was even more profound, with respectively a 4-fold and 12-fold enrichment of KF cells in the BM and spleen (Fig. 4G), confirming previous data describing enrichment of ETP-ALL relapse-inducing cells in the spleen of recipients following venetoclax monotherapy [54]. Altogether, these results show that FLT3 overexpression defines a subset of ETP-ALL repopulating cells highly-resistant to VXL chemotherapy and venetoclax.

### LMO2 establishes autocrine FLT3 signaling in ETP-ALL

**Fig. 5:**
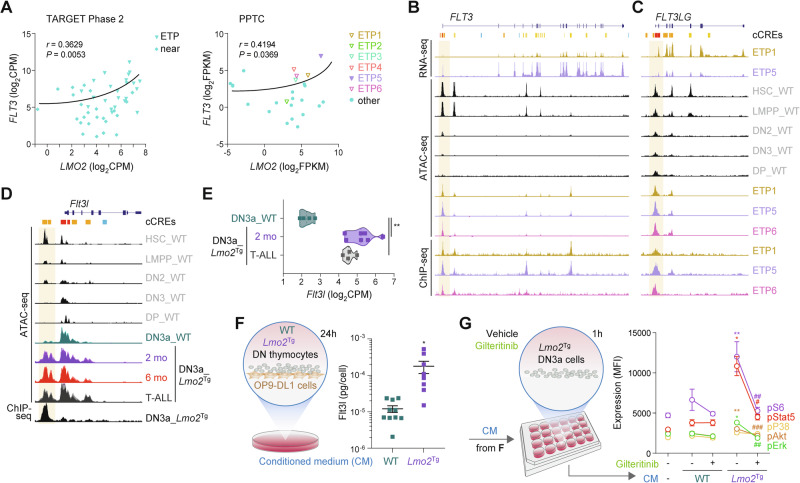
Autocrine FLT3 signaling in *LMO2*-driven ETP-ALL. **A** Correlative studies of *FLT3* and *LMO2* expression in (left) immunophenotypically-defined ETP and near-ETP (near) samples from the TARGET-ALL Phase 2 cohort, and (right) all samples from the PPTC cohort. CPM counts per million mapped reads, FPKM fragments per kilo base of transcript per million mapped fragments, PPTC Pediatric Preclinical Testing Consortium. Pearson correlation coefficient *r* is indicated. Student’s *t*-test. Integrative genomics viewer visualization of the *FLT3* (**B**) and *FLT3LG* (**C**) loci in human ETP-ALL PDX samples. From top to bottom: candidate Cis-regulatory elements (cCREs) are indicated (red = promoter-like signature; orange = proximal enhancer-like signature, blue = CTCF-only); total messenger RNA (mRNA) analyzed by RNA-seq in ETP1 (mustard) and ETP5 (lavender) PDX tumors; chromatin accessibility from publicly available data (gray) in wild-type (WT) hematopoietic stem cells (HSC), FLT3-expressing lymphoid-primed multipotent progenitors (LMPP), DN2 and DN3 T-cell progenitors, DP mature T cells, as well as ATAC-seq data from ETP1 (mustard), ETP5 (lavender) and ETP6 (pink) PDX samples; LMO2 ChIP-seq signals in ETP1 (mustard), ETP5 (lavender) and ETP6 (pink) PDX tumors. **D** Integrative genomics viewer visualization of the *Flt3l* locus in mouse wild-type, preleukemic, and leukemic hematopoietic populations. From top to bottom: candidate Cis-regulatory elements (cCREs) are indicated (red = promoter-like signature; orange = proximal enhancer-like signature, blue = CTCF-only); chromatin accessibility from publicly available data (gray) in long-term HSCs (HSC), DN2a (DN2) and DN3 T-cell progenitors, DP mature T cells, as well as ATAC-seq data from WT (green), 2 month-old *Lmo2*^Tg^ (2 mo; purple), 6 month-old *Lmo2*^Tg^ (6 mo; red), leukemic *Lmo2*^Tg^ (T-ALL; dark gray) DN3a thymocytes; ChIP-seq signals in *Lmo2*^Tg^ DN3a thymocytes. **E** Expression levels of *Flt3l* in WT (green), 2-month-old *Lmo2*^Tg^ (2 mo; purple), and leukemic *Lmo2*^Tg^ (T-ALL; dark gray) DN3a thymocytes. CPM counts per million mapped reads. Median ± S.E.M., ordinary 1-way ANOVA with Tukey’s correction test; **P < 0.01 compared to WT. Levels of **F** Flt3-ligand (Flt3l) in conditioned media (CM) generated from wild-type (WT) or *Lmo2*^Tg^ DN3a thymocytes co-cultured on OP9-DL1 cells for 24 h measured by ELISA, and **G** growth factor-mediated signaling effectors in *Lmo2*^Tg^ DN3a cells stimulated with CM from WT or *Lmo2*^Tg^ DN3a thymocytes for 1 h in presence of vehicle (DMSO) or gilteritinib, assessed by flow cytometry. For ELISA: Student’s *t*-test; *P < 0.05 compared to CM from WT DN thymocytes. For stimulation assays: Mean Fluorescence Intensity (MFI) ± of *N* = 4 individual animals analyzed in duplicate are shown. Mann-Whitney test; *P < 0.05 and **P < 0.01 compared to no stimulation; ^#^*P* < 0.05, ^##^*P* < 0.01 and ^###^*P* < 0.001 compared to vehicle.

Aberrant activation of FLT3 signaling in T-ALL is associated with an ETP immunophenotype and the *LMO2*/*LYL1* subgroup [19]. Bulk transcriptional profiling showed that *FLT3* significantly correlated with *LMO2* expression in ETP/near-ETP samples from both TARGET-ALL Phase2 and the PPTC patient cohorts (Fig. 5A). To assess whether *FLT3* is a direct target of the LMO2 complex, we performed chromatin immunoprecipitation (ChIP)-seq in *LMO2*-expressing ETP-ALL PDX samples. We also performed assay for transposase-accessible chromatin (ATAC)-seq to look for associated opening of chromatin related to the LMO2 binding. Overall, we identified 6,872 LMO2 binding sites shared amongst all three ETP-ALL samples with the majority in CpG open sea regions (63%), intronic and distal intergenic regions (55%; Fig. S9A, B). LMO2 binding was also found within promoter regions (37%; Fig. S9B). Motif enrichment analysis showed enrichment for predicted binding sequences of LMO2 transcriptional partners RUNX1, TAL1, GATA2, ERG, and FLI1 in proximity to LMO2 binding sites (Fig. S9C, D), which were found within the regulatory regions of known LMO2 targets such as *LYL1* and *HHEX* (Fig. S9E, F). We found several LMO2 complex binding sites with differential opening of chromatin (open in HSPCs and closed with normal T-cell differentiation) within *FLT3* promoters and putative intronic enhancers (Fig. 5B). ChIP-seq and ATAC-seq in DN3a thymocytes identified conserved sequences bound by the LMO2 complex with differential opening of chromatin (closed in WT and open in *Lmo2*^Tg^ cells) around the mouse *Flt3* locus (Fig. S9G).

Interrogation of the FLT3-ligand (*FLT3LG*) locus in these human ETP-ALL identified a LMO2 complex binding site within its promoter that also progressively closes during normal T-cell differentiation (Fig. 5C). Similarly, we found a regulatory region bound by LMO2 upstream the *Flt3l* locus associated with differential opening of chromatin in *Lmo2*^Tg^ DN3a thymocytes at all stages of leukemia development (Fig. 5D). Consistent with *Lmo2*-initiated expression, *Flt3l* mRNA levels were increased 7-fold in preleukemic and leukemic *Lmo2*^Tg^ DN3a thymocytes (Fig. 5E), suggesting an autocrine FLT3 signaling loop in *Lmo2*-driven leukemogenesis.

To address whether Lmo2 initiates an autocrine loop in early stages of leukemogenesis, conditioned media (CM) was generated from DN thymocytes isolated from 6-week-old WT and *Lmo2*^Tg^ mice (Fig. 5F). Quantification using ELISA revealed more than 14-fold increased soluble Flt3l production by *Lmo2*^Tg^ T-cell progenitors compared to WT. Next, we tested for the ability of this CM to activate signaling in preleukemic *Lmo2*^Tg^ DN3a cells (Fig. 5G), which display aberrant Flt3 surface expression (Fig. 1B) and respond to in vitro stimulation with Flt3l (Fig. 1D). CM from preleukemic T-cell progenitors induced canonical signaling mediators pStat5, pErk, pS6 and pAkt in *Lmo2*^Tg^ DN3a cells, whilst we observed no activation with CM from WT cells or serum-free fresh medium. Activation of signaling by culture supernatants of preleukemic *Lmo2*^Tg^ T-cell progenitors was completely abrogated by gilteritinib (Fig. 5G), a selective type I FLT3 inhibitor [55–59]. Thus, our data suggest that *LMO2* initiates an autocrine FLT3 signaling early during ETP-ALL development.

### Inhibition of FLT3 signaling induces chemo-sensitivity of LSCs

**Fig. 6:**
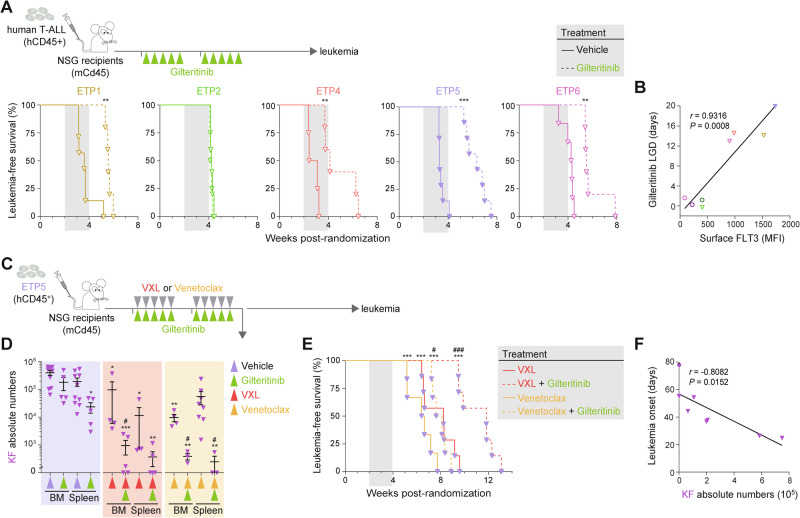
Targeting FLT3 signaling enhances response to chemotherapy in ETP-ALL. **A** Experimental setting for testing the efficacy of gilteritinib monotherapy in several PDX models of human ETP-ALL (top). NSG recipients were randomized and subsequently treated when the average proportion of human leukemic cells reached 1% in the peripheral blood. Kaplan-Meier curves of sublethally-irradiated recipients injected with 5 ETP-ALL PDX models administered with either vehicle or gilteritinib (bottom). Log-rank (Mantel-Cox) test; **P < 0.01 and ***P < 0.0001 compared to vehicle. The period of administration is indicated in light gray. **B** Correlative studies between the gilteritinib-induced leukemia growth delay (LGD) in days and FLT3 expression (MFI). Pearson correlation coefficient *r* is indicated. Student’s *t*-test. **C** Experimental setting for testing the efficacy of gilteritinib, VXL chemotherapy, venetoclax, and combination therapy in the ETP5 xenograft model of human ETP-ALL. NSG recipients were randomized after engraftment was confirmed in the peripheral blood, and subsequently treated when the average proportion of human leukemic cells in the peripheral blood reached 1%. **D** Absolute number of KF tumor cells in the bone marrow (BM) and spleen of ETP5 xenografted recipients, analyzed 24 h after the last drug administration. Mean ± S.E.M., 2-way ANOVA with Tukey correction test; *P < 0.05, **P < 0.01 and ***P < 0.001 as compared to vehicle; ^#^*P* < 0.05 compared to VXL chemotherapy or venetoclax monotherapy. **E** Kaplan-Meier curves of ETP5 xenografted recipients, administered with either vehicle or gilteritinib, in combination with VXL chemotherapy or venetoclax. Log-rank (Mantel-Cox) test; ***P < 0.0001 compared to vehicle (Fig. S10D); ^#^*P* < 0.05 and ^###^*P* < 0.01 compared to VXL chemotherapy or venetoclax monotherapy. The period of administration is indicated in light gray, with the number of recipients for each cohort indicated. **F** Correlation between between the onset of leukemia (days since randomization) in the and the numbers of KF cells in the BM and spleen of ETP5 xenografted recipients treated with either vehicle, gilteritinib, VXL chemotherapy, venetoclax or the combination therapies. Pearson correlation coefficient *r* is indicated. Student’s *t*-test.

We assessed the therapeutic potential of gilteritinib using the different PDX models of ETP-ALL displaying variable levels of FLT3 expression (Figs. 3H and 5A). Daily administration of gilteritinib for 10 days significantly improved survival in all ETP-ALL PDX models except ETP2 (Figs. 6A and S10A), which expressed the lowest levels of FLT3 (Fig. 5A) and did not contain a KF population (Figs. 3G, H and S7B). Furthermore, response to gilteritinib, as defined by leukemia growth delay (LGD) [27, 29, 50], was correlated with bulk FLT3 surface protein and gene expression (Figs. 6B and S10B), suggesting that FLT3 could be used as a predictive marker of response to gilteritinib in ETP-ALL.

Next, we examined the ability of gilteritinib to enhance response to VXL chemotherapy or venetoclax using the ETP5 PDX model (Fig. 6C), which displays the highest FLT3 expression (Fig. 5A) and harbors a FLT3-activating mutation (Fig. S7A) [27]. Twenty-four hours after the last dose of treatment, single agent gilteritinib produced a 90% reduction of total leukemic cells (Fig. S10C), with a similar reduction of KF cells in the BM and spleen (Fig. 6D). Treatment with chemotherapy or single agent venetoclax for 2 weeks produced greater reduction in leukemia burden than gilteritinib (Fig. S10C) but there was relative sparing of KF cells, especially in the spleen of recipients treated with venetoclax (Fig. 6D). The addition of gilteritinib to VXL chemotherapy or venetoclax produced a 99.9% reduction in KF cells. Of note, inhibition of FLT3 signaling effectively targeted the splenic reservoir of venetoclax-resistant cells (Figs. 6D and S10C). Importantly, these combinatorial effects translated into significantly improved long-term survival, especially for recipients that were administered gilteritinib in combination with VXL chemotherapy (Figs. 6E and S10D–F). Decreased numbers of KF cells following treatment correlated with delayed onset of disease (Fig. 6F). Altogether, these results show that gilteritinib can more effectively decrease tumor burden and target treatment-resistant KF cells, thereby improving response to VXL chemotherapy or venetoclax in FLT3-expressing ETP-ALL.

## Discussion

ETP-ALL is characterized by an immature T-cell immunophenotype with aberrant expression of at least one stem and/or myeloid marker [21, 22, 60]. However, the immunophenotypic characterization of LSC populations within the heterogeneous ETP-ALL tumors remains limited [27]. Using a combination of a mouse model of ETP-ALL and patient-derived xenografts, we identified leukemic cells co-expressing KIT and high levels of FLT3 (KF) that were most enriched for leukemia initiating activity, self-renewal, and chemoresistance. Analysis of single cell transcriptomics data from primary human ETP-ALL samples confirmed the presence of rare KF cells (Figs. 3 and S6, 7), which were characterized by interferon/inflammatory response programs known to promote resistance to dexamethasone and other glucocorticoids [61]. Flow cytometry analyses revealed that this KF subpopulation represented a variable fraction of *Lmo2*-driven mouse (Fig. 2G) and *LMO2*-expressing human (Fig. 3H) ETP-ALL tumors. The variable presence of a distinct KF population in ETP-ALL may reflect clonal evolution during disease progression associated with additional lesions, and may explain the failure to identify this population in previous analyses of T-ALL samples harvested at diagnosis and/or relapse [37, 38, 62]. Given the numbers of LSCs may provide prognostic value [5, 12], our findings support adding FLT3 (CD135) to current flow cytometric panels recommended for evaluation of ETP-ALL that could be extended to other T-ALL subtypes.

Both K and KF cells had serial repopulating activity (Fig. 2C) but one distinct and unexpected feature of KF cells was their proliferative nature despite profound resistance to chemotherapy akin to normal HSCs (Figs. 4 and S8B). This observation challenges our previous study of *Lmo2*^Tg^ mice that suggested self-renewal and chemoresistance of LSCs were integrally linked to quiescence [24]. One possible explanation is that KF cells represent a more primate progenitor of K cells, analogous to the switch from actively cycling fetal HSCs to more quiescent adult HSCs [63]. Transplant of K cells could generate KF cells (Fig. S2A), suggesting that the relationship between KF and K cells is a dynamic process rather than lineage commitment. The presence of cycling and dormant LSCs has been described in B-ALL, where the BM endosteum promotes dormancy [13]. A similar process may be occurring in ETP-ALL, where specific niches within the tumor microenvironment provide signals that support proliferation and survival of LSCs including IL-7 [26, 64] and FLT3L [65]. In agreement with this, we observed the progressive expansion of Flt3-responsive KF cell subpopulations during *Lmo2*-driven leukemogenesis (Fig. 2F). Together, our data suggest that aberrant FLT3 signaling promotes progression and recurrence by enhancing the stem-cell-like properties of relapse-inducing cell populations in ETP-ALL.

Activating mutations and aberrant expression of FLT3 is most frequently seen in ETP-ALL [19, 35–38]. The mechanism of increased FLT3 expression in ETP-ALL is unknown but could reflect a proposed ETP cell-of-origin for the disease [21] given that normal ETPs are characterized by FLT3 expression [66–69]. However, mouse models suggest ETP-ALL can arise from more mature T-cell progenitors in which FLT3 expression is progressively silenced during normal differentiation [70–72]. ChIP-seq studies using human ETP-ALL explants identified several binding sites for LMO2 within the regulatory regions of the *FLT3* locus associated with open chromatin that is normally closed in DN3 thymocytes (Fig. 5B). Of note, this promoter LMO2 binding site was conserved in the mouse *Flt3* locus and was associated with open chromatin in all stages of *Lmo2*-driven leukemogenesis (Fig. S9G). Oncogenic activation of FLT3 has been reported for AML and B-ALL [43, 73, 74]. In addition to the *LMO2*-associated expression of FLT3, ChIP-seq identified LMO2 complex binding within a putative regulatory region of the *FLT3LG* locus (Fig. 5C, D), which was associated with aberrant FLT3L expression (Fig. 5C, E). In vitro assays using conditioned media support an autocrine FLT3/FLT3L signaling loop (Fig. 5F, G). More detailed studies of the LMO2 binding sites in regulating regions of *FLT3* and *FLT3LG* loci will be needed but our findings provide the first evidence for a mechanistic link between LMO2 and FLT3 signaling in ETP-ALL.

FLT3 inhibitors have transformed the therapeutic landscape for *FLT3*-mutated AML [75] with single agent gilteritinib more effective than chemotherapy for relapsed disease [56, 58, 59, 76, 77]. Somewhat surprisingly, testing FLT3 inhibitors in ETP-ALL, where the frequency of FLT3-activating mutations is up to 35%, has progressed little since in vitro studies that demonstrated activity [38, 78]. Our results demonstrate single agent efficacy that was correlated with bulk *FLT3* expression in tumors (Figs. 6B and S10B). We show that gilteritinib prevents the downstream activation of multiple canonical signaling pathway mediators including pStat5 (Fig. 5G), which is an essential target of LSCs in ETP-ALL [27]. Thus, targeting pStat5 may also explain the ability of gilteritinib to overcome LSC chemoresistance. Given chemotherapy has been shown to increase expression of *FLT3LG* [65], pre-treatment or delaying gilteritinib until after chemotherapy may be even more effective. In addition to current clinical trials incorporating JAK inhibitors, our pre-clinical results support testing of FLT3 inhibitors for ETP-ALL with high FLT3 expression and/or FLT3-activating mutations.

Activating mutations of FLT3 are more common in adult and relapsed ETP-ALL, where intensive chemotherapy may not be tolerated. BCL2 inhibition is an attractive approach for older or less fit individuals with ETP-ALL, which express high levels of BCL2 [79–81]. Several clinical trials are testing the BCL2 inhibitor venetoclax combined with chemotherapy or demethylating agents in ETP-ALL (ClinicalTrials.gov identifier: NCT03319901, NCT03826992; Chinese Clinical Trial Register: ChiCTR2200061708, ChiCTR2100048966). FLT3-mediated signaling promotes resistance to the selective BCL2 inhibitor venetoclax in relapsed/refractory AML [82, 83]. Conversely, combination with gilteritinib has been shown to synergize with venetoclax in AML, irrespective of the FLT3 mutation status [77, 82–84]. Here we extend these findings by providing pre-clinical evidence that gilteritinib enhanced the efficacy of venetoclax in a relevant PDX model of human ETP-ALL (Figs. 6D, E and S10C–F), which included significant reduction of the splenic reservoir of venetoclax-resistant leukemic cells [54]. Synergy may be explained by gilteritinib inhibiting key signaling mediators of resistance to venetoclax (e.g., STAT5, PLK1, LCK) [85–87]. Our results are similar to the combination of venetoclax and the MERTK inhibitor MRX-2843, which also inhibits FLT3 [88]. However, the advantage of gilteritinib is the proven track record in *FLT3*-mutated AML including combination with venetoclax [77].

In conclusion, our work identified FLT3 overexpression as a potential immunophenotypic marker for chemoresistant LSCs in *LMO2*-driven ETP-ALL. In accordance with previous reports of selective efficacy of FLT3 inhibitors in *MLL*-rearranged ALL with high levels of FLT3 [89], we provide compelling in vivo data validating bulk FLT3 expression as a predictive biomarker for molecular response to gilteritinib in ETP-ALL irrespective of FLT3 mutation status. Together, these data provide a cellular, and molecular explanation for enhanced cytokine signaling in *LMO2*-driven ETP-ALL beyond activating mutations, and a rationale for inclusion of type I FLT3 inhibitors as a new precision medicine approach for FLT3-expressing ETP-ALL.

## Methods

### Mouse experiments using genetic models

All experiments were performed in accordance with the relevant guidelines and regulations, and pre-approved by the Alfred Research Alliance (ARA) Animal Ethics Committee and the Animal Care Committee (ACC) at the Bannatyne campus, University of Manitoba. The current study was performed by using the previously described *CD2-Lmo2* (*Lmo2*^Tg^) [90] and *TetOP-H2B-GFP*^KI/+^(*H2B-GFP*) [24, 91] mouse models. All mouse lines were backcrossed onto a C57BL/6J background for 10 generations and maintained in pathogen-free conditions according to institutional animal care guidelines.

### Flow cytometry analyses

Flow cytometry analysis and cell sorting were done as previously described [25, 30] on single cell suspensions of T-cell progenitors. Cell cycle analysis was performed as described previously [30], using an antibody against Ki67 (1:10; BD Pharmingen, Cat#556027) or the isotype control, and staining DNA using 4’,6-diamidino-2-phenylindole (DAPI, Sigma-Aldrich). Analyses were performed using LSRII and LSR Fortessa cytometers and cell sorting was performed with a FACSAria or BD Influx (BD Pharmingen). A complete list of antibodies used is provided in Supplementary Material.

### Isogenic murine transplantation assays

Transplantation assays were performed by intravenously injecting thymus cells into at least 3 sublethally-irradiated (550 Rads) isogenic Ly5.1 (Cd45.1) mice. Fold expansion was calculated by dividing the absolute output number of donor-derived DN3a cells harvested at the experimental endpoint by the respective number of DN3a thymocytes injected in each recipient. Limiting dilution transplantations were performed as described [92], by injecting various doses (10^1^ to 10^6^) of thymocytes per recipient, and mice were scored positive when T-cell lineage reconstitution assessed 6 weeks after transplantation was greater than 1%. Preleukemic stem cell (preLSC) frequency was calculated by applying Poisson statistics using the ELDA: Extreme Limiting Dilution Analysis software (Walter and Eliza Hall Institute Bioinformatics, Parkville VIC, Australia) [93]. Limiting dilution transplantations were performed independently from any other transplantation assays, using thymocytes from 3 different *Lmo2*^Tg^ mice for each cohort. For in vivo studies with primary *Lmo2*^Tg^ T-ALL, we used the sample size equation: *N* = 2*s*^*2*^((Za/2+Zb)^2^)/(*un*-*uo*)^2^ where standard deviation *s*, observed difference *un*-*uo*, power of 0.8, and significance of *p* < 0.05 (Za/2 + Zb)^2^ = 7.85. With an average proportion of 6.4 ± 2.5% of leukemic KF cells in T-ALL 027 (Fig. 4E) a variation of 6% would be biologically significant and thus, we require 2 × (2.5)^2 ^× 7.85/(6)^2^ = 3 recipients per treatment arm. Leukemic mice were scored positive when the proportion of T-ALL (Cd45.2^+^) cells in the peripheral blood reached 50%. *Kaplan-Meier* survival and statistical analysis were performed using *GraphPad* Prism 8.0 software (*GraphPad* Software Inc, San Diego, CA).

### Serial transplantation assays

For primary transplantation, 2 × 10^6^ thymocytes were intravenously injected into at least 5 sublethally-irradiated (550 Rads) isogenic Ly5.1 (Cd45.1) mice. For subsequent transplantations, 5 × 10^6^ thymocytes harvested from recipients were subsequently injected into sublethally-irradiated Cd45.1^+^ mice, as previously described [24].

### In vivo tracking of cell-cycle kinetics

Assessment of cell-cycle kinetics using the *TetOP-H2B-GFP*^KI/+^;*Lmo2*^Tg^ (*H2B-GFP*;*Lmo2*^Tg^) mouse model was performed as previously described [24, 28]. Briefly, thymocytes were enumerated after 6 weeks of Doxycycline pulse, followed by 2 weeks of chase. Proportion of bulk or subpopulations of DN3a cells retaining the H2B-GFP label (GFP^hi^) was analyzed using flow cytometry.

### Single-cell RNA-seq analysis

Human ETP-ALL single-cell RNA-seq data was obtained from publicly deposited data (GSE161901) [48]. Clustering of single-cell profile and gene expression analyses were performed using Seurat [94] and MSigDB database [95]. Cell type annotation was performed using CIPR R package from ImmGen [49, 96]. Gene Ontology (GO) enrichment of differentially-expressed genes (DEGs; Wilcoxon rank test, average log_2_FC > 0.25 and adjusted *p* < 0.01).

### VXL chemotherapy

All drugs were resuspended in PBS 1X prior to intraperitoneal administration at a daily dose of 0.15 mg/kg for vincristine (vincristine sulfate injection; Pfizer Australia), 5 mg/kg for dexamethasone (DBL^TM^ dexamethasone sodium phosphate injection, Pfizer Australia) and 1000 U/kg for *L*-Asparaginase (ENZ-287, ProSpec-Tany TechnoGene Ltd).

### RNA-seq analysis

Total RNAs for global gene expression were extracted from 0.5 to 2 × 10^5^ sorted cells from individual mice as previously described [24]. Extracted RNA was analyzed for concentration and quality using the 2200 Tapestation Analyzer (Agilent Technologies). mRNA-focused sequencing libraries were then generated using the NEBNext Ultra II Directional RNA Library Prep Kit v3.1_5/20 (Illumina). Sequencing of samples were performed on a NovaSeq 6000 SP Reagent Kit (Illumina). TARGET-ALL Phase 2 data was obtained from Genomic Data Commons Data Portal using TCGAbiolinks (2.18.0) [97, 98]. Data from the PIVOT Pediatric Preclinical Testing Consortium (PPTC) was obtained from the PedcBioPortal for Cancer Genomics [47].

### ATAC-seq analysis

ATAC-seq was performed from 50,000 FACS-sorted cells using Tagmenting mix (Illumina Australia Pty Ltd, Australia) and amplification of transposed DNA fragments [99]. Public HSC, LMPP, DN2, DN3 and DP data was obtained from ImmGen project (GSE100738) [72]. Analysis was performed as previously described [100].

### ChIP-seq analysis

ChIP was performed using either mouse thymocytes or human ETP-ALL cells with the LMO2 monoclonal antibody (AF2726, R&D Systems). Libraries were generated using the TruSeq sample preparation kit (Illumina) and sequenced on a NextSeq 500 (Illumina). HiSeq2000 analyzer (BGI, Hong Kong). Data analysis was performed using custom pipeline 10.5281/zenodo.6371682.

### In vitro autocrine signaling assays

For generating conditioned medium, 10^6^ lineage-depleted CD4^−^CD8^−^ (DN) thymocytes were seeded onto OP9-DL1 stromal cells (kindly provided by Dr. Juan-Carlos Zúñiga-Pflücker) [39], and co-cultured for 24 h in reconstituted alpha-MEM medium (12561, Gibco, Life Technologies) supplemented with 10% heat-inactivated FBS (12318, Gibco), 10 mM HEPES (15630-060, Gibco), 1 mM sodium pyruvate (11360-070, Gibco), 55μM β-mercaptoethanol (21985-023, Gibco), 2 mM Glutamax (15750-060, Gibco), Penicillin/Streptomycin (15140-122, Gibco), 5 ng/mL IL-7 (217-17, PeproTech, Rock Hill NJ, USA), as previously described [101]. After 24 h of co-culture, the conditioned media were collected, and Flt3l levels were assessed by ELISA using the Mouse Flt3 Ligand DuoSet ELISA (DY427, R&D Systems) and the DuoSet ELISA Ancillary Reagent Kit 2 (DY008B, R&D Systems). For stimulation assays, conditioned media was harvested and centrifuged at 300 × *g* for 5 min, with 200μl distributed in a 96-well V-bottom microtest plate (82.1583001, Sarstedt) containing 100,000 sorted DN3a thymocytes from 6-week-old *Lmo2*^Tg^ mice in presence of either dimethyl sulfoxide (DMSO; D2438, Sigma-Aldrich) or 10 nM gilteritinib (S7754, Selleckhem) diluted in DMSO. Cytokine-mediated response in DN3a thymocytes was assessed by flow cytometry after incubation for an hour at 37 °C.

### Pre-clinical assays with patient-derived xenografts (PDXs)

PDX models were established as previously described [102, 103]. All experiments were performed in accordance with the relevant guidelines and regulations, and pre-approved by the Human Research Ethics Committee of the University of New South Wales (UNSW), the Alfred Health Human Ethics Committee, the ARA Animal Ethics Committee, and the Bannatyne campus ACC committee of the University of Manitoba. Informed consent was obtained from all subjects. For transplantation assays, splenocytes harvested from successfully engrafted primary human ETP-ALL or T-ALL xenografts were injected into nonobese diabetic/ severe combined immunodeficient NOD/SCID/*Il2rg*^tm1wjl^/SzJ (NSG) mice [104]. For in vivo studies with PDX models, we used the sample size equation: N = 2*s*^2^((Za/2 + Zb)^2^)/(*un*-*uo*)^2^ where standard deviation *s*, observed difference *un*-*uo*, power of 0.9, and significance of *p* < 0.05 (Za/2 + Zb)^2^ = 10.51. With an average proportion of 2.6 ± 0.4% of KF cells in ETP5 tumors from the BM (Fig. 4G) a variation of 1% would be biologically significant and thus, we require 2 × (0.4)^2 ^× 10.51/(1)^2^ = 4 recipients per treatment arm. Engraftment was monitored by flow cytometry analyses of peripheral blood as previously described [27, 29]. Blinded from the investigator, NSG recipients were randomized after engraftment was confirmed in the peripheral blood, and treatment commenced when the average proportion of human leukemia cells in the peripheral blood reached 1%. Both male and female recipients were used. Gilteritinib and venetoclax were administered daily by oral gavage for 5 consecutive days a week for 2 weeks at a dose of 10 mg/kg and 50 mg/kg, respectively [105, 106]. Tumor burden was assessed at 24 h after the last dose of drug was administered, as previously described [27, 29]. *Kaplan-Meier* survival and statistical analysis were performed using *GraphPad* Prism. 6.0 software (*GraphPad* Software Inc, San Diego, CA).

## Supplementary information

Supplementary Material

## Data Availability

The datasets generated during the current study are available in the Gene Expression Omnibus (GEO) repository. All relevant data that support the findings of this study are available from the corresponding author upon reasonable request.

## Code availability

All relevant codes and/or references to these codes that were used to generate the data featured in this study are available from the corresponding author upon reasonable request.
