# Supplementary material for: Targeting LMO2-induced autocrine FLT3 signaling to overcome chemoresistance in early T-cell precursor acute lymphoblastic leukemia
Source: Leukemia. 2025 Jan 23;39(3):577–89. doi: 10.1038/s41375-024-02491-5 (PMC11879882; doi:10.1038/s41375-024-02491-5)
Supplement: Supplementary file 1 — Supplementary Material [file 41375_2024_2491_MOESM1_ESM.docx]

**Targeting *LMO2*-induced autocrine FLT3 signalling to overcome chemoresistance in early T-cell precursor acute lymphoblastic leukemia**

Tremblay CS, *et al*.

**SUPPLEMENTARY FIGURES…………………………………………………...…………..……………2**

Figure S1. *Lmo2*-associated changes within the DN3 T-cell progenitor population.….……………...2

Figure S2. Flt3 expression associated with proliferation and expansion of preLSCs……..…..…..…3 Figure S3. Transcriptional profiling of human T-ALL samples……………………………………….….5

Figure S4. Cluster-defining markers in human ETP-ALL samples…………………..………..….…….6

Figure S5. Cell type annotations in human ETP-ALL samples………………………….………………7

Figure S6. Molecular programs in KF cells from ETP-ALL patient samples…………………………..8

Figure S7. Transcriptional profiling of ETP-ALL PDX models………………………….……………….9

Figure S8. Preleukemic and leukemic KF cells are more resistant to chemotherapy ……..……….10

Figure S9. *LMO2*-driven transcriptional regulation of FLT3 and FLT3L in ETP-ALL..………..…….12

Figure S10. Efficacy of gilteritinib in patient-derived ETP-ALL xenografts…......…………...……….14

**SUPPLEMENTARY METHODS………...………………………………………………………....…….16**

**SUPPLEMENTARY REFERENCES………………………………………………...………......………18**

**
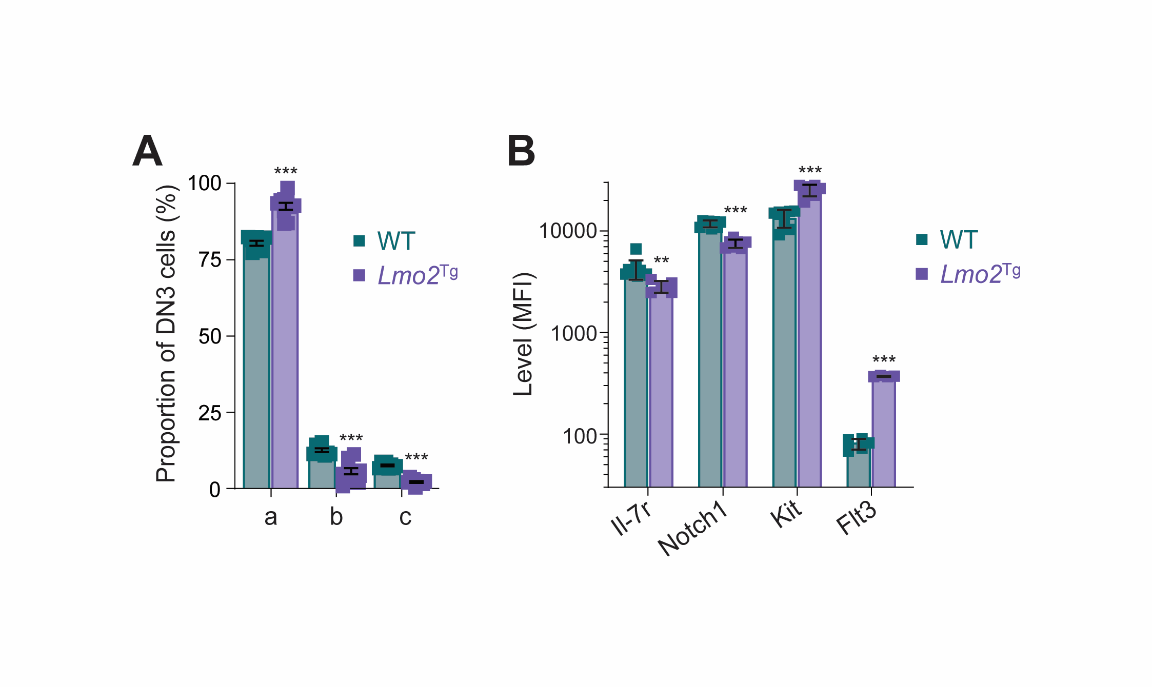
Supplementary Figures**

**Figure S1. *Lmo2*-associated changes within the DN3 T-cell progenitor population.** Proportion of the different subsets (a, b, c) of DN3 thymocytes (**A**), and levels of Il-7r (CD127), Notch1, Kit (CD117) and Flt3 (CD135) at the surface of DN3a T-cell progenitors (**B**), from 6-week old WT and *Lmo2*-transgenic (*Lmo2*^Tg^) mice. Mean fluorescence intensity (MFI) ± S.E.M., 2-way ANOVA with Tukey’s correction test; ***P*<0.01 and ****P*<0.001 compared to WT controls.

**
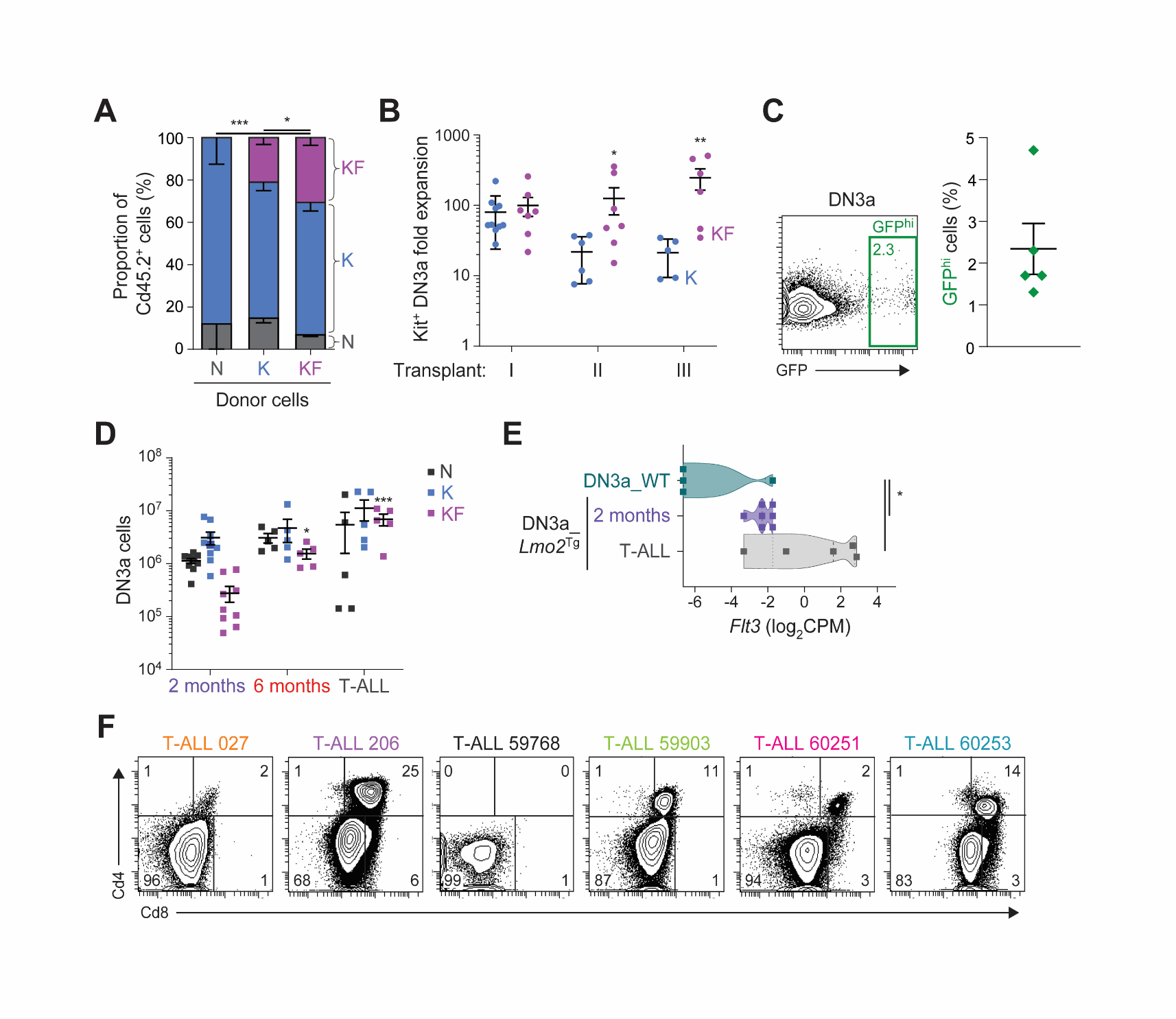
Figure S2. Flt3 expression associated with proliferation and expansion of preLSCs. A,** Immunophenotype of donor-derived N, K and F populations of DN3a thymocytes enumerated in the thymus of primary recipient mice. Median ± S.E.M., 2-way ANOVA with Tukey’s correction test; **P*<0.05 compared to K donor cells, ****P*<0.001 compared to N donor cells. **B,** Fold expansion of donor-derived *Lmo2*^Tg^ K and KF subpopulations to generate KIT^+^ DN3a cells in the thymus of primary (I), secondary (II) and tertiary (III) recipients. Mean ± S.E.M., 2-way ANOVA with Tukey’s correction test; **P*<0.05 compared to K donor cells. **C,** Representative flow cytometric analysis of GFP expression (left), and quantification of cells retaining high levels of GFP labelling (GFP^hi^; right), in DN3a thymocytes from *H2B-GFP; Lmo2*^Tg^ mice after 6 weeks of labelling with Doxycycline followed by 2 weeks of chase. Profile: GFP^hi^ populations are framed, with the average proportion (%) indicated. Quantification: Mean ± S.E.M. **D,** Absolute numbers of DN3a T-cell subpopulations enumerated in the thymus of *Lmo2*^Tg^ mice at 6- and 12-month of age and at overt T-ALL. Mean ± S.E.M., 2-way ANOVA with Tukey’s correction test; **P*<0.05 and ****P*<0.001 compared to 2-month-old *Lmo2*^Tg^ mice. **E,** Expression levels of *Flt3* in WT (green), 2-month-old *Lmo2*^Tg^ (purple), and leukemic *Lmo2*^Tg^ (T-ALL; dark grey) DN3a thymocytes. CPM: counts per million mapped reads. Median ± S.E.M., ordinary 1-way ANOVA with Tukey’s correction test; **P*<0.05 compared to WT. **F**, Representative flow cytometry analysis of Cd4 and Cd8 expression in primary *Lmo2*^Tg^ leukemias. Average proportion of Cd4^+^, Cd8^+^, Cd4^+^Cd8^+^ (DP) and Cd4^-^Cd8^-^ (DN) populations are indicated for each T-ALL.

**
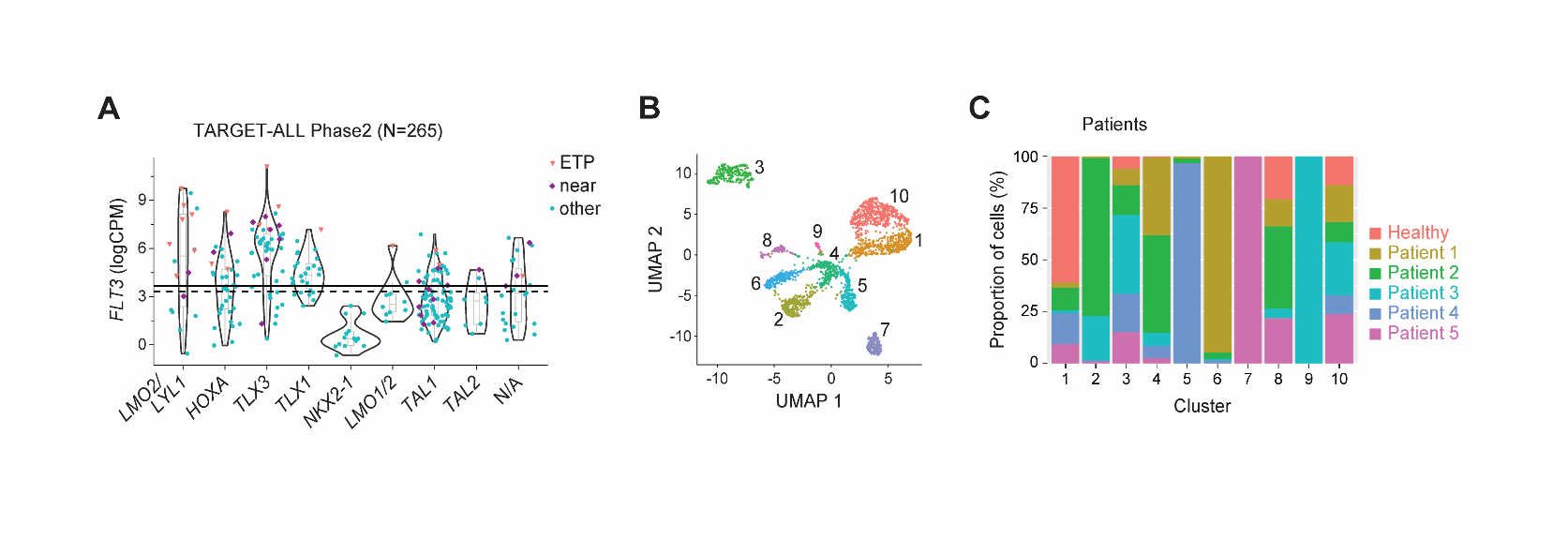
Figure S3. Transcriptional profiling of human T-ALL samples. A,** Expression levels of *FLT3* in primary T-ALL samples from the TARGET-ALL Phase 2 cohort. Oncogene-defined subclasses of T-ALL listed with N/A including other uncharacterized samples. Immunophenotypes are indicated including ETP-ALL (ETP), near-ETP (near) and other more mature T-ALL subtypes (other). CPM: counts per million mapped reads. Solid line: mean; dashed line: median. **B,** Two-dimension uniform manifold approximation and projection (UMAP) of the processed single-cell RNA-seq gene expression data from 4-5 healthy individuals and 5 patients with refractory/relapsed ETP-ALL visualized in color-coded clusters. **D,** Donor origin of each cluster of the processed single-cell RNA-seq gene expression data from healthy individuals and patients with refractory/relapsed ETP-ALL. Proportion (%) of each origin is color-coded to identify individual samples.

**
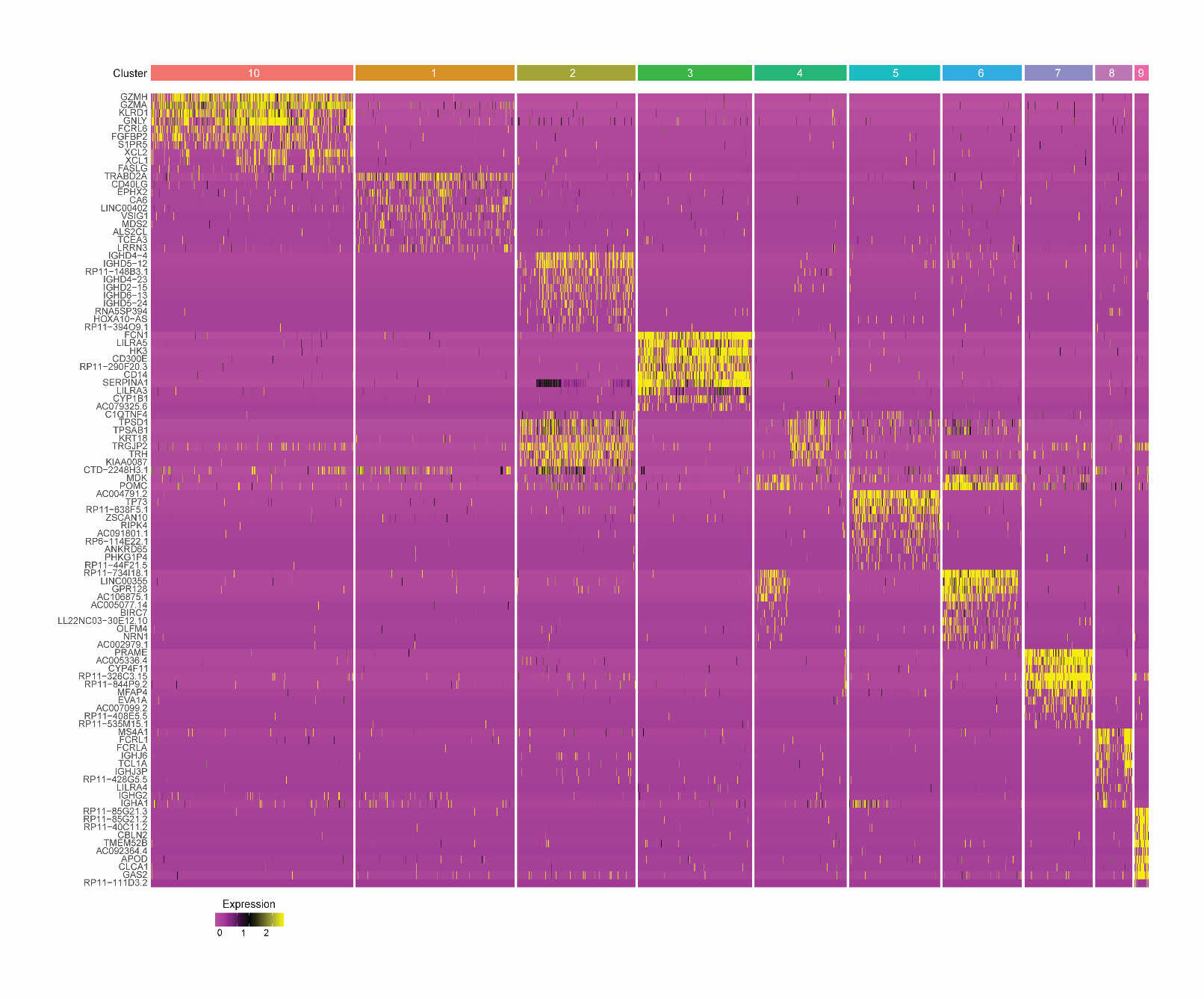
Figure S4. Cluster-defining markers in human ETP-ALL samples.** Heatmap of the expression for the top 10 markers for each cluster generated from the processed single-cell RNA-seq gene expression data from 5 patients with refractory/relapsed ETP-ALL.

**
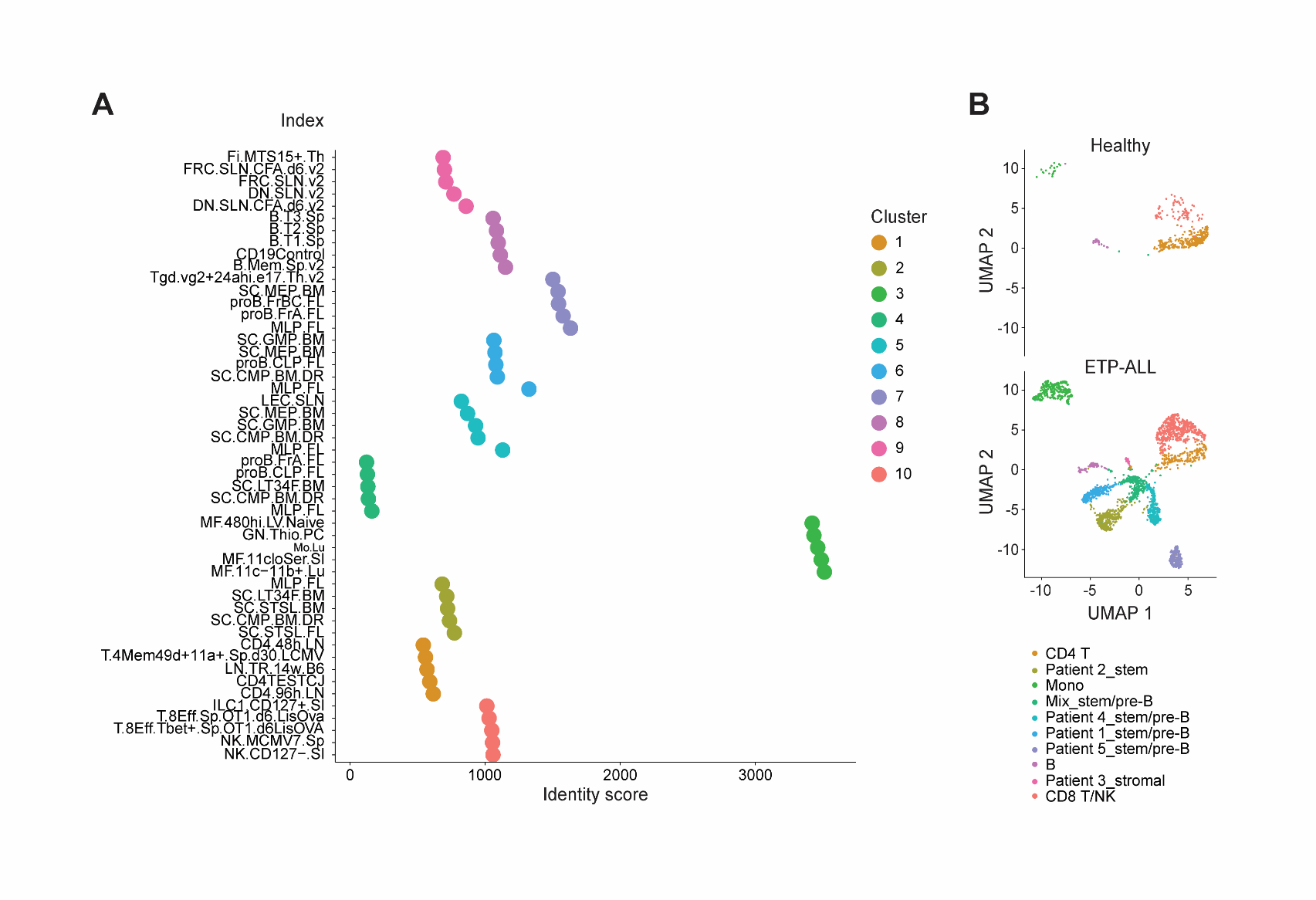
Figure S5. Cell type annotations in human ETP-ALL samples.**  **A**, Predicted cell type based on marker genes of each cluster determined using ImmGen^1^ as a reference. **B,** Two-dimension uniform manifold approximation and projection (UMAP) of the clusters labelled using origin and cell type annotations for healthy individuals and 5 patients with refractory/relapsed ETP-ALL**.**

**
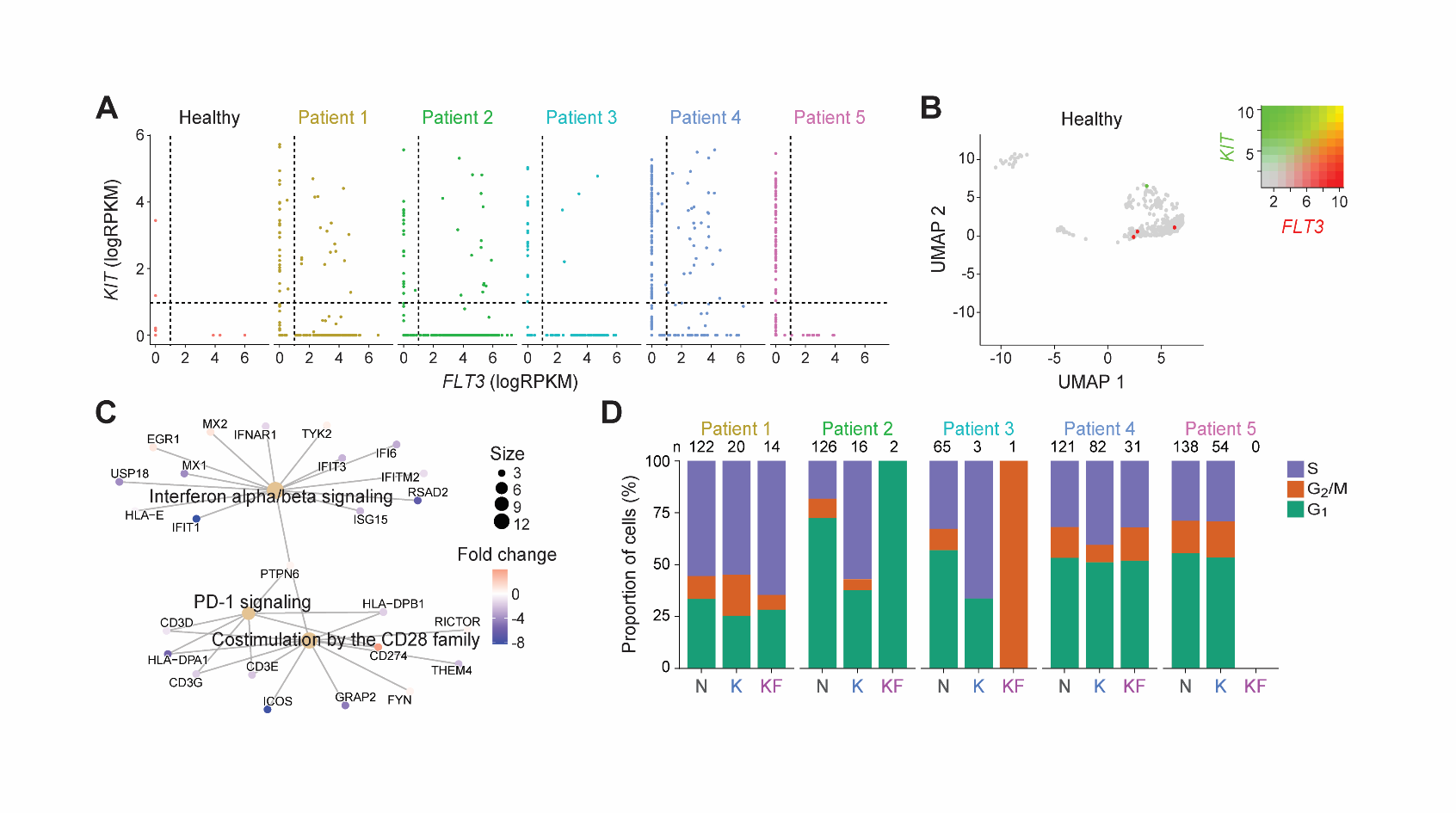
Figure S6. Molecular programs in KF cells from ETP-ALL patient samples. A,** Log-normalized *FLT3* and *KIT* expression in individual ETP-ALL patients. RPKM: reads per kilo base of transcript per million mapped reads. Dashed line: cut-off of 1 used to define subpopulations. Number of cells analyzed per sample: Patient 1 (N=222), Patient 2 (N=272), Patient 3 (N=114), Patient 4 (N=258) and Patient 5 (N=198). **B,** Two-dimension uniform manifold approximation and projection (UMAP) of log-normalized *FLT3* and *KIT* expression in processed single-cell RNA-seq data from 4-5 healthy individuals (merged). Expression of KIT in green and FLT3 in red, with co-expression in yellow (KF cells). **C,** Reactome pathways enrichment from the top 1,000 differentially-expressed genes (DEGs) in KF compared to K cells from all ETP-ALL patients analyzed. The size of the circle represents the number of genes enriched in the pathway, the color of the circle represents the adjusted *P*-value. **D,** Proportion of N, K and KF cells in each phase of the cell cycle from single-cell RNA-seq analysis from individual ETP-ALL samples. Numbers of cells in each subpopulation indicated. Color-coded cell cycle phases, with G_1_ (green), S (purple) and G_2_/M (orange).

**
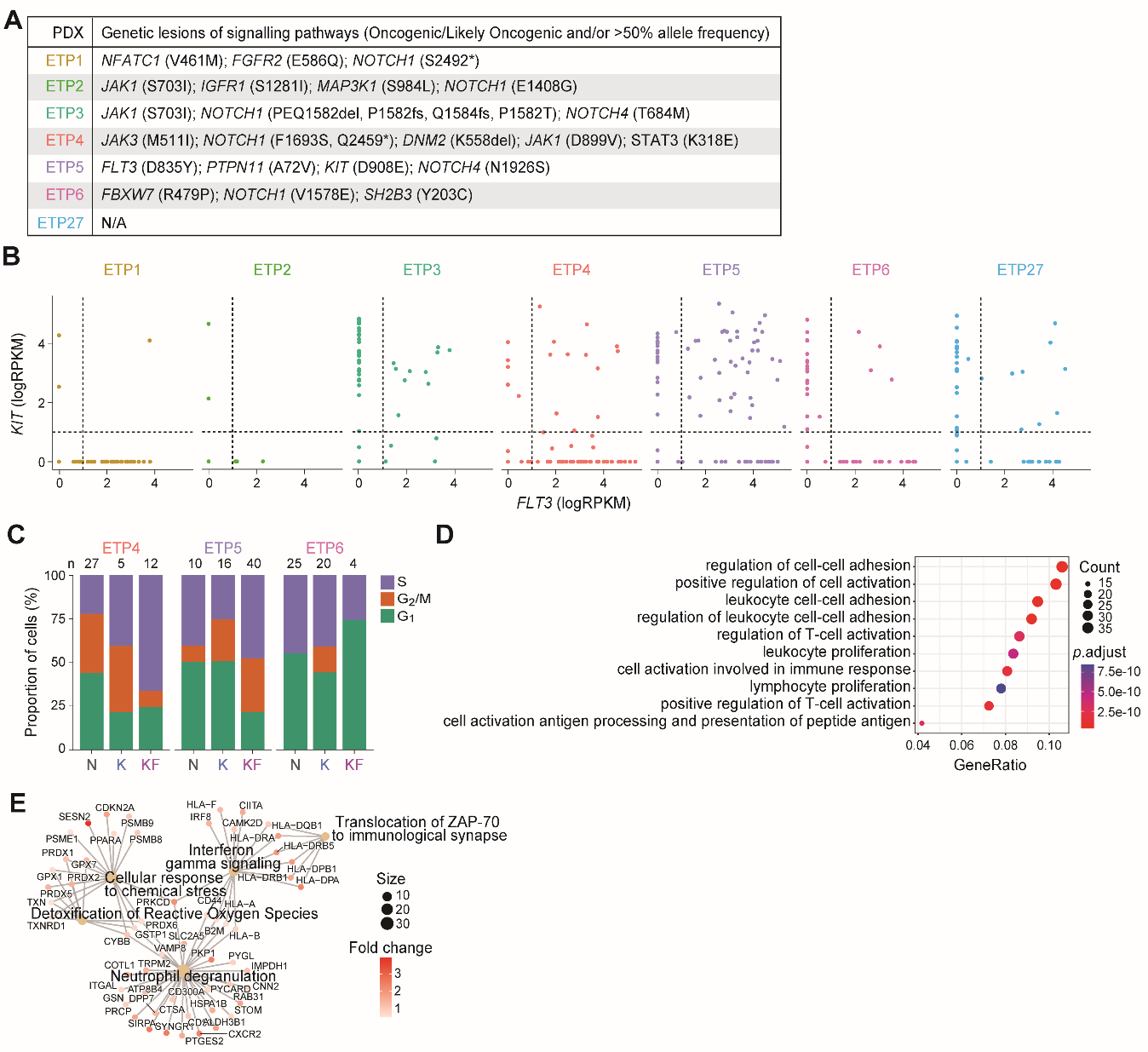
Figure S7. Transcriptional profiling of ETP-ALL PDX models.** **A,** Mutations of signalling pathways for each patient-derived ETP-ALL xenograft. The amino-acid position for each mutation is shown, fs means frameshift, del means deletion. Complete list of mutations in each PDX previously reported^2, 3^. **B,** Log-normalized *FLT3* and *KIT* expression in individual ETP PDX samples. RPKM: reads per kilo base of transcript per million mapped reads. Dashed line: cut-off of 1 used to define subpopulations. Number of cells analyzed per sample: ETP 1 (N=92), ETP2 (N=55), ETP3 (N=50), ETP4 (N=82), ETP5 (N=82), ETP6 (N=64) and ETP27 (N=66). **C,** Proportion of cell-cycle phase for N, K and KF cells from individual ETP PDXs analyzed. Numbers of cells in each subpopulation indicated. Color-coded cell cycle phases, with G_1_ (green), S (purple) and G_2_/M (orange). **D-E,** Gene Ontology (GO) enrichment of biological processes (**D**), and enriched Reactome pathways (**E**), from the top 500 DEGs in KF compared to all other tumor cell populations from ETP PDX samples. The size of the circle represents the number of genes enriched in the pathway, the color of the circle represents the adjusted *P*-value.

**
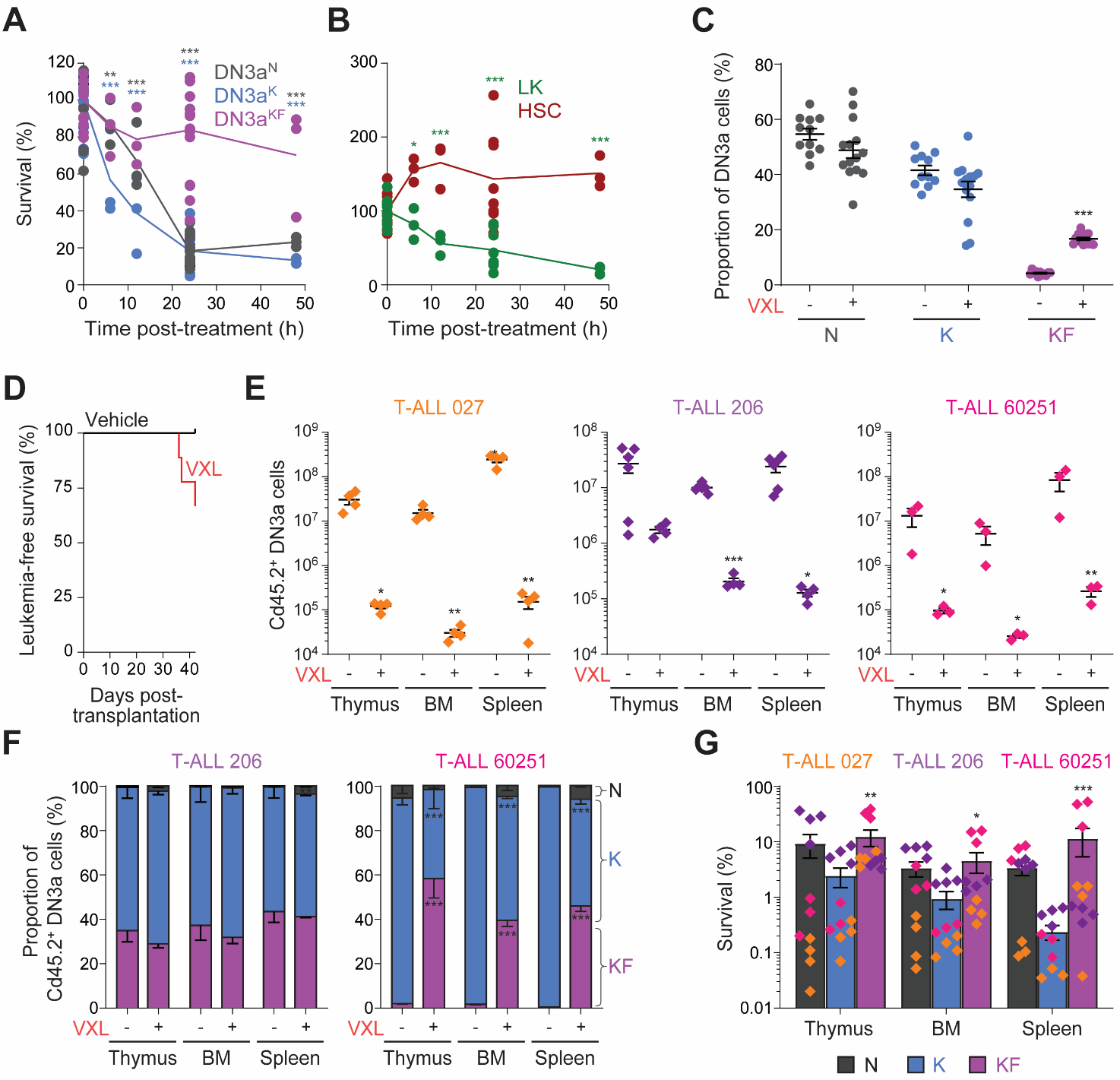
Figure S8.** **Preleukemic and leukemic KF cells are more resistant to chemotherapy. A-B,** Survival of the DN3a T-cell progenitor subpopulations (N, K and KF) in the thymus (left), as well as HSCs (Lin^-^Sca-1^+^Kit^+^CD150^+^CD48^-^) and Lin^-^Kit^+^ (LK) progenitors in the bone marrow (right) of 6-week-old *Lmo2*^Tg^ mice, at the indicated time points following administration of VXL chemotherapy. Median (solid line), Mean ± S.E.M., 2-way ANOVA with Tukey’s correction test; **P*<0.05, ***P*<0.01, ****P*<0.001 compared to baseline (t=0). **C,** Proportion of the different DN3a thymocyte subpopulations from 6-week old *Lmo2*^Tg^ mice, assessed by flow cytometry 24 hours after the last administration of VXL chemotherapy. Mean ± S.E.M., 2-way ANOVA with Tukey’s correction test; ****P*<0.001 compared to baseline (Vehicle). **D,** Kaplan-Meier curves of mice injected with DN3a thymocytes harvested 24h after the last administration of either Vehicle or VXL chemotherapy (black: 10^4^ Vehicle-treated DN3a^KF^ cells N=9; red: 10^4^ VXL-treated DN3a^KF^ cells, N=9). **E-F,** Absolute numbers of donor-derived (Cd45.2^+^) DN3a leukemic cells (**E**), and proportion (%) of subpopulations of N, K and KF cells (**F**) in the thymus, bone marrow (BM) and spleen of recipients transplanted with primary *Lmo2*^Tg^ T-ALL (T-ALL 027, T-ALL 206 and T-ALL 60251), assessed by flow cytometry 24 hours after the last administration of VXL chemotherapy. Mean ± S.E.M., 2-way ANOVA with Bonferroni correction test; **P*<0.05, ***P*<0.01, ****P*<0.001 as compared to vehicle. **G,** Survival of the DN3a T-cell progenitor subpopulations in the indicated hematopoietic organs of recipients, at 24h following administration of vehicle or VXL chemotherapy. Median ± S.E.M., ordinary 1-way ANOVA with Tukey’s correction test; ****P*<0.001 compared to other subpopulations.

**
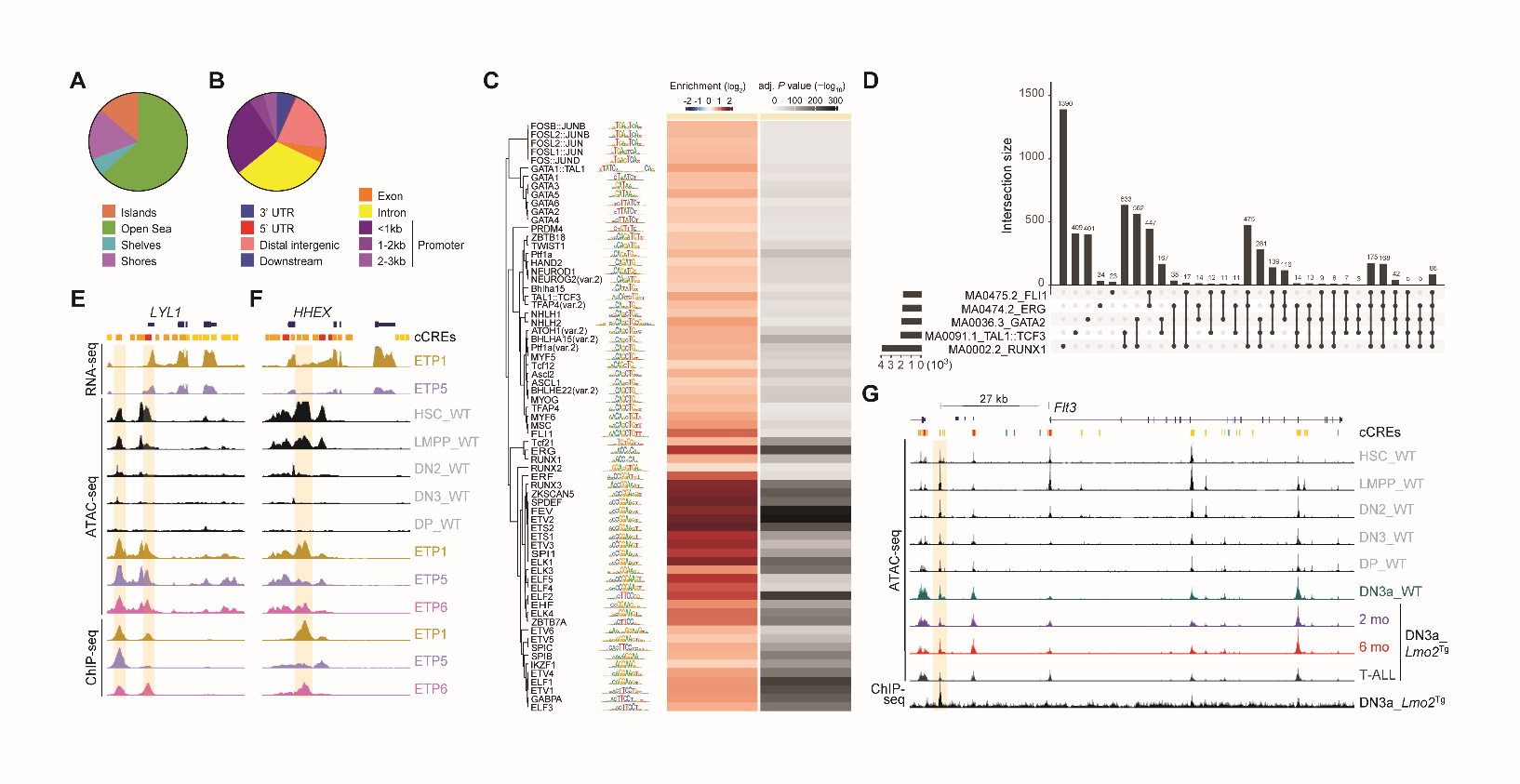
Figure S9. *LMO2*-driven transcriptional regulation of FLT3 and FLT3L in ETP-ALL. A-B,** Genomic location of LMO2 binding defined by ChIP-seq with regions were divided into (**A**) promoter CpG islands, surrounding shores (within 2 kb of CpG islands), shelves (2-4 kb from promoter CpG

islands) and open sea (all other regions), or (**B**) 3’ untranslated regions (UTR), 5’UTR, distal intergenic, downstream, exon, intron or promoter regions (<1kb, 1-2kb, 2-3kb from initiation site). **C-D,** Motif enrichment analysis has identified ETS, GATA, E box, RUNX and KLF/SP motifs (**C**), which appeared in combinations at LMO2-binding sites (**D**). Enrichment (log2) and adjusted *P*-values are listed for each binding motif. Upset plot comparing the peak overlap between FLI1, ERG, GATA2, TAL1::TCF3 and RUNX1 transcription partners, including possible combinations between the different partners (dot plot), the number of peaks bound by each combination (vertical bar plot), and the total number of peaks bound by each transcription factor (horizontal bar plots; 10^3^). **E-F,** Integrative genomics viewer visualization of the *LYL1* (**E**) and *HHEX* loci (**F**). From top to bottom: candidate Cis-regulatory elements (cCREs) are indicated (red = promoter-like signature; orange = proximal enhancer-like signature, blue = CTCF-only); total messenger RNA (mRNA) analyzed by RNA-seq in ETP1 (mustard) and ETP5 (lavender) PDX tumors; chromatin accessibility from publicly available data (grey) in wild-type (WT) hematopoietic stem cells (HSC), FLT3-expressing lymphoid-primed multipotent progenitors (LMPP), DN2 and DN3 T-cell progenitors, DP mature T cells, as well as ATAC-seq data from ETP1 (mustard), ETP5 (lavender) and ETP6 (pink) PDX samples; LMO2 ChIP-seq signals in ETP1 (mustard), ETP5 (lavender) and ETP6 (pink) PDX tumors. **G,** Integrative genomics viewer visualization of the *Flt3* locus. From top to bottom: candidate Cis-regulatory elements (cCREs) are indicated (red = promoter-like signature; orange = proximal enhancer-like signature, blue = CTCF-only); chromatin accessibility from publicly available data (grey) in long-term HSCs (HSC), DN2a (DN2) and DN3 T-cell progenitors, DP mature T cells, as well as ATAC-seq data from WT (green), 2 month-old *Lmo2*^Tg^ (2 mo; purple), 6 month-old *Lmo2*^Tg^ (6 mo; red), leukemic *Lmo2*^Tg^ (T-ALL; dark grey) DN3a thymocytes; ChIP-seq signals in *Lmo2*^Tg^ DN3a thymocytes.

**
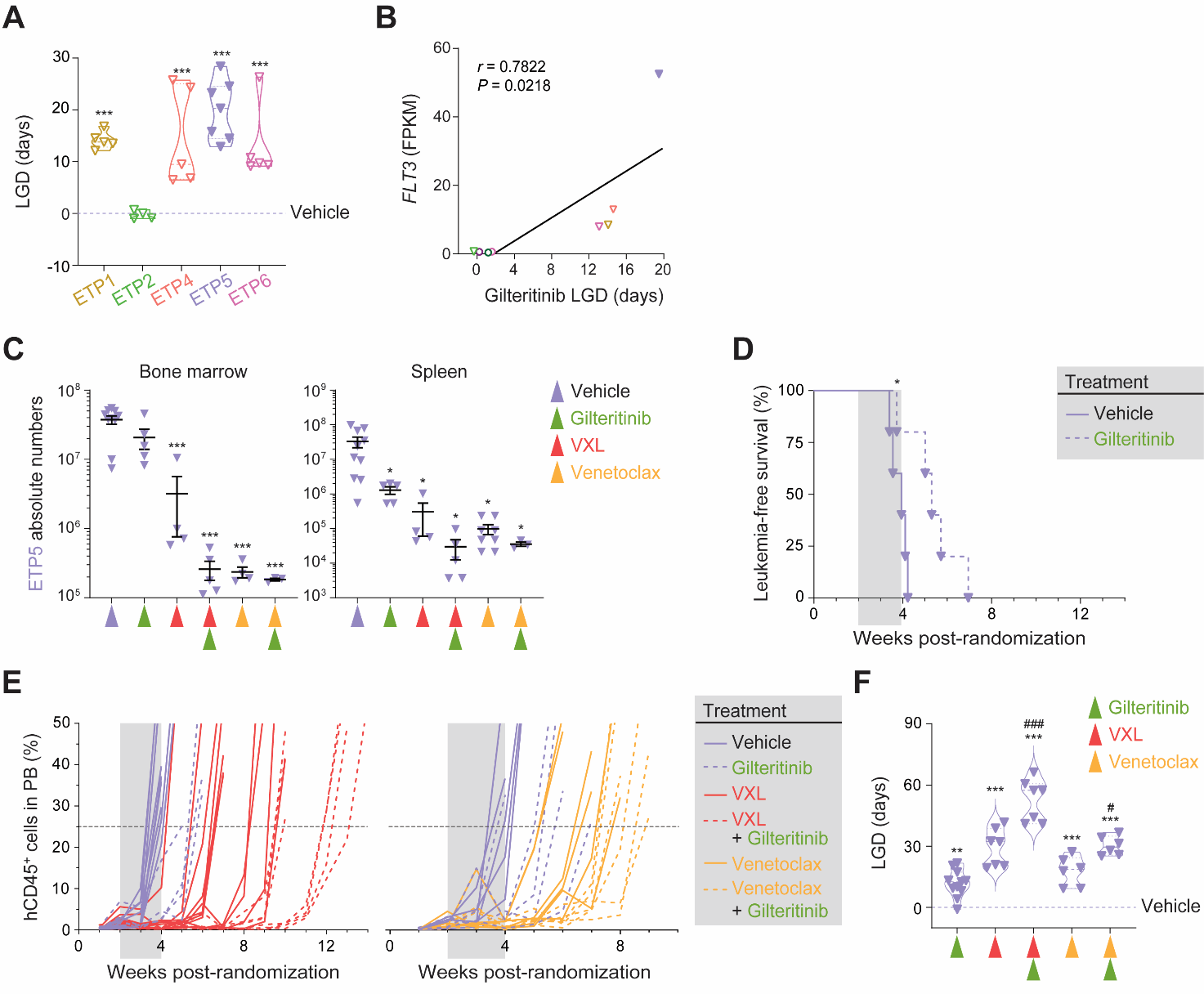
Figure S10. Efficacy of Gilteritinib in patient-derived ETP-ALL xenografts. A,** Leukemia growth delay (LGD) in recipients injected with the indicated ETP-ALL PDX models treated with gilteritinib as a single agent. Dashed lines within the violin represents the 25^th^, 50^th^ and 75^th^ quartiles, 2-way ANOVA with Tukey’s correction test; ****P*<0.001 as compared to vehicle. **B,** Correlative studies between the gilteritinib-induced leukemia growth delay (LGD) in days and levels of FLT3 mRNA transcripts (FPKM: fragments per kilo base of transcript per million mapped). Symbols of individual ETP PDX models as indicated in **A**. Pearson correlation coefficient *r* is indicated. Student’s *t*-test. **C,** Absolute number of ETP5 cells in the bone marrow and spleen of recipients xenografted with ETP5, analyzed 24h after the last drug administration. Mean ± S.E.M., 2-way ANOVA with Tukey correction test; **P*<0.05 and ****P*<0.001 as compared to vehicle. **D**, Kaplan-Meier curves of ETP5 xenografted recipients, administered with either vehicle or gilteritinib as a single agent. Log-rank (Mantel-Cox) test; **P*<0.05 compared to vehicle. The period of administration is indicated in light grey. **E**, Proportion of patient-derived leukemic cells (%hCD45^+^) in the peripheral blood (PB) of recipients xenografted with ETP5, treated with gilteritinib, VXL (left), venetoclax (right) or combination therapy. The period of administration is indicated in light grey. **F,** Leukemia growth delay (LGD) in recipients injected with ETP5 treated with gilteritinib as a single agent, VXL chemotherapy, venetoclax and combination therapy. Dashed lines within the violin represents the 25^th^, 50^th^ and 75^th^ quartiles, 2-way ANOVA with Tukey’s correction test; ***P*<0.01, ****P*<0.001 as compared to vehicle; ^#^*P*<0.05, ^###^*P*<0.001 compared to VXL or venetoclax alone.

**Supplementary Methods**

**Flow cytometry.** Flow cytometry analyses and sorting were performed as previously described^4-6^ on single-cell suspensions stained using BD Pharmingen antibodies against mouse CD4 (RM4-5), CD8 (53-6.7), CD25 (PC61.5), CD44 (IM7), CD45.1 (A20), CD45.2 (104), Thy1.2 (53-2.1; #561616), TCRβ (H57-597), CD117 (ACK45), CD135 (A2F10.1), CD150 (TC15-12F12.2), B220 (RA3-6B2), CD3 (145-2C11), CD19 (ID3), Gr-1 (RB6-8C5), CD11b (M1/70), Ter119 (Ter119), Sca-1 (D7), and an eBioscience antibody (eBioscience, Invitrogen) against mouse Notch1 (22E5). DN3 populations and Il-7r were stained using BioLegend antibody (Australian Biosearch, Balcatta WA, Australia) against mouse CD28 (E18) and CD127 (A7R34), respectively.

Phosphoflow analysis was performed as previously described^6, 7^. Briefly, 2x10^6^ cells stained for surface markers were subsequently resuspended into PBS 1X + 4% v/v PFA, and incubated for 10 minutes at 4^o^C for fixation. Fixed cells were washed twice using PBS 1X, then resuspended in pre-chilled at -20^o^C Perm Buffer III (BD Pharmingen) and incubated 30 minutes at 4^o^C for permeabilization. Cells were washed twice using PBS 1X, and incubated overnight at 4^o^C in PBS 1X + 2% v/v FCS with Cell Signalling purified antibodies (Genesearch Pty Ltd, Arundel QLD, Australia) against phospho-STAT5 (Tyr694; #9359), phospho-p38 MAPK (Thr180/Tyr182; #4511), phospho-p44/42 (Erk1/2; #9102), phospho-S6 (Ser235/236; #4856) and phospho-Akt (Ser473; #9271) or isotype control. The next day, stained cells were incubated in permeabilization buffer with donkey Alexa Fluor 488-conjugated (A-21206, lot #1910751, Molecular Probes, Invitrogen) or goat Alexa Fluor 546-conjugated (A-11035, lot #1904467) anti-rabbit secondary antibodies for 1 h on ice, and washed twice in cold PBS 1X.

Cell cycle analysis was performed as described previously^4^, using an antibody against Ki67 (1:10; BD Pharmingen, Cat#556027) or the isotype control, and staining DNA using 4',6-diamidino-2-phenylindole (DAPI, Sigma-Aldrich). Analyses were performed using LSRII and LSR Fortessa cytometers and cell sorting was performed with a FACSAria or BD Influx (BD Pharmingen).

Flow cytometry analyses on human T-ALL cells were done on single cell suspensions, as previously described^3, 6^. Briefly, 2x10^6^ cells from patient-derived ETP-ALL xenografts were stained for surface markers using BD Pharmingen antibodies against human CD45 (HI30; #557748), CD135 (4G8; #563908) and BioLegend antibody against human KIT (A3C6E2), washed twice in cold PBS 1X + 2% v/v FCS, then fixed and permeabilized using the BD Cytofix/Cytoperm^TM^ Kit (#554714, BD Australia). Finally, cells were stained using a BioLegend antibody against human cytoplasmic CD3 (HCHT1). FACS analysis was performed using LSRII and LSR Fortessa cytometers.

**Supplementary References**

1. Heng TS, Painter MW, Immunological Genome Project C. The Immunological Genome Project: networks of gene expression in immune cells. *Nature immunology* 2008 Oct; **9**(10)**:** 1091-1094.

2. Anand P, Guillaumet-Adkins A, Dimitrova V, Yun H, Drier Y, Sotudeh N*, et al.* Single-cell RNA-seq reveals developmental plasticity with coexisting oncogenic states and immune evasion programs in ETP-ALL. *Blood* 2021 May 6; **137**(18)**:** 2463-2480.

3. Tremblay CS, Saw J, Boyle JA, Haigh K, Litalien V, McCalmont HR*, et al.* STAT5 activation promotes progression and chemotherapy-resistance in early T-cell precursor acute lymphoblastic leukemia. *Blood* 2023 Mar 29.

4. Tremblay M, Tremblay CS, Herblot S, Aplan PD, Hebert J, Perreault C*, et al.* Modeling T-cell acute lymphoblastic leukemia induced by the SCL and LMO1 oncogenes. *Genes & development* 2010 Jun 1; **24**(11)**:** 1093-1105.

5. McCormack MP, Young LF, Vasudevan S, de Graaf CA, Codrington R, Rabbitts TH*, et al.* The Lmo2 oncogene initiates leukemia in mice by inducing thymocyte self-renewal. *Science* 2010 Feb 12; **327**(5967)**:** 879-883.

6. Tremblay CS, Chiu SK, Saw J, McCalmont H, Litalien V, Boyle J*, et al.* Small molecule inhibition of Dynamin-dependent endocytosis targets multiple niche signals and impairs leukemia stem cells. *Nature communications* 2020 Dec 4; **11**(1)**:** 6211.

7. Tremblay CS, Brown FC, Collett M, Saw J, Chiu SK, Sonderegger SE*, et al.* Loss-of-function mutations of Dynamin 2 promote T-ALL by enhancing IL-7 signalling. *Leukemia* 2016 May 13.
